# Removal and elimination of pharmaceuticals in water using zeolites in diverse adsorption processes and catalytic advanced oxidation technologies—a critical review

**DOI:** 10.1007/s11356-024-35204-7

**Published:** 2024-11-04

**Authors:** Efraím A. Serna-Galvis, Johana Arboleda-Echavarría, Adriana Echavarría-Isaza, Ricardo A. Torres-Palma

**Affiliations:** 1 Grupo de Investigación en Remediación Ambiental y Biocatálisis (GIRAB), Instituto de Química, Facultad de Ciencias Exactas y Naturales, Universidad de Antioquia UdeA, Calle 70 # 52-21, Medellín, Colombia; 2 Grupo de Catalizadores y Adsorbentes (CATALAD), Instituto de Química, Facultad de Ciencias Exactas y Naturales, Universidad de Antioquia UdeA, Calle 70 # 52-21, Medellín, Colombia; 3 Escuela de Microbiología, Universidad de Antioquia UdeA, Calle 70 # 52-21, Medellín, Colombia

**Keywords:** Aluminosilicates, Catalytic ozonation, Fenton process, Persulfate activation, Pollutants transformation, Water treatment

## Abstract

Water pollution by pharmaceuticals is a current worrying environmental problem. Adsorption and catalytic processes using zeolites have been employed in several studies to remove/degrade pharmaceuticals from water. The interest of researchers in these two strategies based on the utilization of zeolites (i.e., adsorption and advanced oxidation technologies, AOT) is continuously growing. Then, this work presents a literature review, considering the origin of the zeolites (natural vs. synthetic) and the modifications of zeolites (e.g., the addition of surfactants) for the adsorption of diverse pharmaceuticals. The role of zeolites in catalytic ozonation, Fenton-based systems, and activation of peroxymonosulfate and peroxydisulfate is detailed. Also, the primary transformations of pharmaceuticals induced by these AOTs were examined. Moreover, the gaps regarding biodegradability and toxicity of the transformation products coming from the degradation of pharmaceuticals by the zeolites-based processes were discussed. To overcome the scarcity of information regarding the biodegradability and toxicity of the primary transformation products observed in the revised works, an initial approach to these topics, using a predictive tool, was made. Finally, from the present review, it was evidenced the need for future works involving zeolites that provide results about the simultaneous removal/elimination of multiple pharmaceuticals in complex matrices (e.g., hospital wastewater or municipal wastewater), new information about biodegradability and toxicity plus the development of combination or coupling of processes with other AOTs (e.g., sonochemistry) or classical systems (e.g., biological process).

## Introduction

For the last three decades, pharmaceuticals have been recognized as environmental pollutants of emerging concern (Küster and Adler 2014; Patel et al. 2019). Despite these substances being ubiquitous, persistent, and biologically active, currently, most countries around the world have incipient requirements for the discharge of pharmaceuticals into the environment (Verlicchi et al. 2012; Patel et al. 2019). Pharmaceuticals differ from other water pollutants in features such as their complex chemical structures, multiple ionization groups, and polymorphism. Moreover, the use of pharmaceuticals is not exclusive to humans, they are also utilized in animal farming on a large scale. For instance, animal antibiotics use far surpasses human consumption. After consumption, a portion of the ingested pharmaceuticals is excreted and ends up in the environment (Patel et al. 2019).

Biological activity is a particular problem with pharmaceuticals in the environment. Pharmaceuticals can elicit physiological changes in organisms even at low concentrations. Scientific reports have shown that pharmaceuticals can induce toxic effects from molecular (e.g., inhibition of cyclooxygenases) to population levels (e.g., behavioral changes, effects on reproduction). For instance, it is well-known that hormones such as ethinyl estradiol contribute to the feminization of male fish downstream from sewage treatment plants (Larsson et al. 2007; Küster and Adler 2014). Also, the continuous exposure of microorganisms and humans to antibiotics in wastewater effluents can contribute to the evolution of drug-resistant bacteria (Patel et al. 2019).

Considering the above-mentioned concerns about pharmaceuticals, several researchers have focused their works on diverse biological, physical, or chemical processes to remove/degrade these contaminants from aqueous samples (Patel et al. 2019). Among such processes, the adsorption of pollutants on zeolites and the utilization of zeolites in advanced oxidation technologies (AOTs) are areas of growing interest due to the high efficacy of these materials to act as adsorbents and catalysts for the removal and degradation of organic pollutants in water (Hussain et al. 2020; Kohantorabi et al. 2021; Araújo et al. 2021; Issaka et al. 2022; Jalali et al. 2022).

Some previous reviews have been focused on the adsorption of metals (Yuna 2016) and the removal of organic pollutants such as dyes, even by using AOTs (Jiang et al. 2018; Hussain et al. 2020), but none review has been focused on the removal/elimination of pharmaceuticals throughout adsorption and AOTs based on the utilization of zeolites. Hence, this work aimed to review the topic of the adsorption on zeolites of highly consumed pharmaceuticals such as analgesics (e.g., ibuprofen, diclofenac), antibiotics (sulfonamides, fluoroquinolones), or antihypertensives (e.g., atenolol), considering the origin of the zeolites (natural vs. synthetic). Besides, the modifications of zeolites (e.g., addition of surfactants), the role of zeolites in three representative AOTs (catalytic ozonation, Fenton-based systems, and activation of persulfates (i.e., peroxymonosulfate, and peroxydisulfate)) is detailed. Also, the primary transformations of pharmaceuticals induced by these AOTs were examined. Moreover, special attention was paid to the gaps in the processes based on the use of zeolites, regarding biodegradability and toxicity of the transformation products coming from the degradation of pharmaceuticals in aqueous samples. Finally, considering the scarcity of biodegradability and toxicity information in the revised works, an initial contribution to overcoming these lacking topics was made in this review using a free online predictive tool.

## Adsorption of pharmaceuticals on zeolitic materials

### Natural and synthetic zeolites

**Fig. 1:**
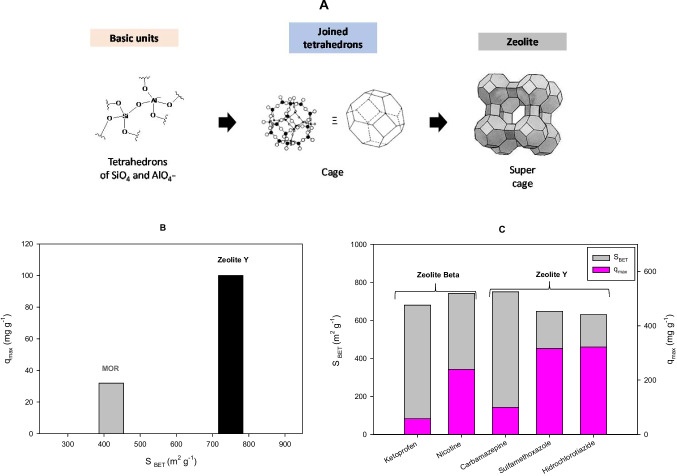
**A** Representation of a zeolite structure (the pore space inside zeolites is parceled into cages and channels. Cages, also named cavities, are the polyhedral units while channels in zeolites are conformed by linked polyhedral units. The channels vary from straight to sinusoidal shapes or from wide to narrow. Many adsorption-related properties of zeolites, e.g., specific surface area (S_BET_), are decided by the structure of cages and channels (Jiang et al. 2018)). **B** Effect of specific surface area (S_BET_) on the maximum adsorption capacity (*q*_max_) of zeolites having the same SiO_2_/Al_2_O_3_ ratio (200) adsorption of carbamazepine (figure done by the review authors considering information presented in the reference Martucci et al. (2012)). **C** Adsorption of pharmaceuticals on zeolites has large micropore volumes (figure done by the review authors considering information presented in the references Martucci et al. (2012), Pasti et al. (2013), Blasioli et al. (2014), Jiang et al. (2018), and Sarti et al. (2020))

Zeolites are defined as crystalline inorganic polymers consisting of tetrahedra (Król 2020). They are based on corner-sharing tetrahedral structures (TO_4_, T: mainly silicon and aluminum), forming three-dimensional networks with regularly sized pores (Cataldo et al. 2021) (Fig. 1A). In the zeolite structure, the silicon tetravalent (SiO_4_) has a neutral charge, while the aluminum trivalent units are negatively charged (AlO_4_^−^). Because of the Al^3+^ presence in the network, zeolites have easily exchangeable compensation cations (e.g., Na^+^, K^+^, or Ca^2+^); also, water molecules can be trapped in their pores (Yuna 2016). Furthermore, the networks of TO_4_ form regular three-dimensional structures (crystals), which follow specific patterns that give rise to the zeolite framework types, having the well-known three-letter code nomenclature (e.g., FAU, MFI, BEA) (Katzer 1989).

As zeolites have open channels (pores) in their structure, this provides space for the adsorption of diverse organic molecules and zeolites also act as molecular sieves (Cataldo et al. 2021; Feng et al. 2021). Hence, zeolites have important uses in environmental protection. Indeed, some zeolites have a high selectivity for the removal of water pollutants such as heavy metal ions and ammonium ions (Król 2020). They can also be utilized as catalysts for degrading environmental pollutants (Kohantorabi et al. 2021; Jalali et al. 2022). Zeolites can be categorized according to their origin into natural and synthetic materials. Natural zeolites come from volcanic sources mainly, and they are commonly formed at low-temperature and low-pressure alteration between aqueous fluid reactions with rocks (Szerement et al. 2021). Natural zeolites are found in crystallized forms in igneous and metamorphic rocks (filling vugs and fractures) and in sedimentary rocks as grains of smaller diameters. Moreover, the sediments in the ocean bottom are rich in zeolites, and tuffs or clays can also contain these kinds of minerals. Natural zeolites are varied in structure, composition, and properties. Clinoptilolite, mordenite, and chabazite are the most common naturally occurring zeolites (Król 2020; Szerement et al. 2021). Natural zeolites have advantages such as low cost of obtention and attainability. The chemical composition of a natural zeolite varies and depends on its source place (Szerement et al. 2021).

Zeolites are microporous materials, and another classification of zeolites is based on the pores size being extra-large-pore zeolites with free pore diameters of 0.8–1.0 nm, large-pore zeolites with a free pore diameter of 0.6–0.8 nm, medium-pore zeolites with free pore diameters of 0.45–0.6 nm, and small-pore zeolites with a free pore diameter of 0.3–0.45 nm (Cataldo et al. 2021). Many natural zeolites belong to the small pore size category (which is the case of clinoptilolite, the most common zeolite in nature). This is a disadvantage because natural zeolites do not allow for the adsorption of large inorganic and organic compounds. Besides, the deposits of natural zeolites are non-renewable resources, and the formation process takes tens to thousands of years in natural conditions. Additionally, their applications as catalysts are reduced because the pores are quickly obstructed, and consequently, a rapid deactivation of the material is generated. For these reasons, it is important to synthesize zeolites that allow obtaining materials with specific characteristics for practical applications.

Synthetic zeolites are those produced in a laboratory under controlled conditions. Such zeolites are also diverse in structure, composition, and properties (indeed, the variety of synthetic zeolites is much higher than those of natural origin). A key difference between natural and synthetic zeolite consists of the compositional aspect. Natural zeolites contain predominantly Si and Al (and to a minor extent B and Be) as framework cations, while synthetic ones have a big diversity of framework cations: Si, Al, P, Ge, B, Zn, Ga, Fe, Li, Co, Mg, and Mn and even Ti, Cu, Cd, V, and Cr (all these elements can form tetrahedra with oxygen). Regarding the non-framework constituents, the main difference between natural and synthetic zeolites is the fact that synthetic zeolites can contain as the compensation ions, organic molecules (e.g., amines), or cations exclusively or combined with inorganic cations (Marler and Gies 2018).

Most synthetic zeolites, in quantity and variety, are generated by applying hydrothermal methods. Such syntheses are mostly made at a specific temperature in the range of 80–240 °C under pressurized and closed systems (e.g., autoclave vessels) to allow the nucleation and growth of the zeolite crystals (Marler and Gies 2018). Due to the elevated amount involved in the synthesis process, time is a determinant for the crystal ordering and phase stability. For instance, a synthetic zeolite (made in the laboratory quickly) may exhibit different properties to a structure-related natural zeolite (which takes a long geological time for its formation) due to a dissimilar ordering (Clifton 1987).

Because of the higher speed of the synthetic processes compared to the natural ones, many synthesized zeolites (presenting non-equilibrium or metastable phases) do not have natural zeolite counterparts. In laboratory synthesis of zeolites, the Si/Al molar ratio is a critical parameter, and even under similar conditions, small differences in such ratio can lead to completely different zeolites (Clifton 1987). It is relevant to mention that independently of natural or synthetic origin, both kinds of zeolites have adsorbing and catalytic properties that are useful to remove and degrade pharmaceuticals in water samples (such topics are detailed in the subsequent sections).

### Some characteristics of the zeolites employed for the adsorption of pharmaceuticals

An advantage of synthetic zeolites over natural ones is that the formers can be obtained with larger pore sizes, allowing the sorption of big molecules (e.g., pharmaceuticals) without blocking their pores; also, they remain more stable for a longer time in catalytic reactions (Król 2020). On the other hand, natural zeolites are obtained in mixtures with different minerals and impurities, which makes it necessary to carry out pre-treatments for the partial or total reduction of impurities before their use in environmental applications. In this sense, synthetic zeolites have the additional advantage of being obtained with lower impurities (Souza et al. 2023).

From a compositional point of view, both natural and synthetic zeolites can also be classified according to their ratio of Si and Al in the framework. The ratio of Si and Al, also known as the silica-to-alumina ratio, is typically measured as either the Si/Al mole ratio or the SiO_2_/Al_2_O_3_ mole ratio. It must be taken into account that the Si/Al ratio is two times the SiO_2_/Al_2_O_3_ mole ratio (Jiang et al. 2018). The Si/Al ratio (or equivalently the SiO_2_/Al_2_O_3_ ratio) is used to describe the hydrophobicity of the zeolite surface, and this is a determinant parameter for the adsorption of pollutants such as pharmaceuticals on zeolites.

According to the Si/Al ratio, the zeolites can be categorized as high siliceous (Si/Al ratio ≥ 10), intermediate (Si/Al ratio from 2 to 5), and aluminous ones (Si/Al ratio between 1.0 and 1.5). Also, it should be considered that zeolites have both Brønsted and Lewis acidities. A Brønsted acid is any species that can donate an H^+^, whereas a Lewis acid is a species that can accept a pair of electrons. In zeolites, Brønsted acid sites are related with H bridged to Si–O–Al groups (oxygen framework) while the Lewis acid ones are induced by the metal cations (Feng et al. 2021). The increase in the Si/Al ratio reduces the total acidity and increases the hydrophobicity of zeolites. Indeed, siliceous zeolites are organophilic toward non-polar substances (as several pharmaceuticals), while aluminous ones are strong desiccant agents (Cataldo et al. 2021; Feng et al. 2021).

We should mention that the applications of zeolites in water treatment are strongly influenced by the Si/Al ratio. Low-silica zeolites (i.e., Si/Al ratio < 2) are very good for ion exchanges. Because of the high presence of negative charges around Al sites in low-silica zeolites, these can be used for water softening, removal of ammonium, and adsorption of heavy metals (e.g., zinc, nickel, copper, and cadmium) in aqueous samples. Besides, this class of zeolites has also exhibited strong adsorption capability toward cationic organic pollutants. Meanwhile, high-silica zeolites (so-called hydrophobic zeolites) show favorable characteristics for the adsorption of neutral and anionic organic pollutants in aqueous solutions, due to such zeolites having a limited number of cations and negative charges around Al sites, and most of the framework structures remain neutral. It must be said that FAU, MOR, MFI, and BEA types are high-silica zeolites commonly used for the adsorption of organic pollutants (e.g., pharmaceuticals) (Jiang et al. 2018). Also, it can be mentioned that clinoptilolite (the most common zeolite in nature) typically has a Si/Al ratio higher than 4 (Ferri et al. 2024).

In addition to the Si/Al ratio, surface area or micropore volume (characteristics that are determined by the cages and channels of zeolites) influence the adsorption properties (Jiang et al. 2018). For instance, Fig. 1B compares two zeolites with the same Si/Al ratio but different specific surface areas (S_BET_), having higher pollutant adsorption on the zeolite with the larger surface area. Moreover, Beta or Y zeolites, which have large S_BET_, have shown high capacities for the adsorption of diverse pharmaceuticals, as illustrated in Fig. 1C.

### Adsorption of pharmaceuticals from water using non-modified zeolites

One of the pioneer works on the adsorption of pharmaceuticals from water on zeolites was developed by Ötker and Akmehmet-Balcıoğlu (Ötker and Akmehmet-Balcıoğlu 2005). Therein, the adsorption of enrofloxacin (a veterinary antibiotic belonging to the fluoroquinolone class) on the natural zeolite clinoptilolite was presented, showing that the adsorption interactions of enrofloxacin with clinoptilolite are controlled by ionic binding between the antibiotic and zeolite due to the presence of charged moieties on the adsorbent and the adsorbate. Thus, the pH of the solution strongly influences the adsorption process because the charges on the zeolite surface and the pharmaceutical are modified as the pH changes. At acidic pH (5.0), enrofloxacin is positively charged and undergoes cation exchanges, and at neutral pH (7.0), when the pharmaceutical is a zwitterion, the cationic moiety has a large contribution to enrofloxacin adsorption. At basic pH (10), the pharmaceutical enrofloxacin is anionic, and its adsorption is decreased because of repulsion charges with the zeolite (which has a net negative structural charge in the pH range of 2.0–12.0, resulting from isomorphic substitutions of cations in the crystal lattice). Indeed, cation bridging (i.e., the interaction between anionic enrofloxacin and exchangeable cations bonded to negative charges on the surface of the natural zeolite) is proposed to explain the adsorption of anionic enrofloxacin (Ötker and Akmehmet-Balcıoğlu 2005).

The adsorption of pharmaceuticals from water using natural zeolites has also been applied for the removal of the analgesic acetylsalicylic acid and the antihypertensive atenolol (Rakić et al. 2013); the antibiotics moxifloxacin and norfloxacin (Rubashvili et al. 2019); the ibuprofen, diclofenac, paracetamol, and indomethacin analgesics (Al-rimawi et al. 2019); the antipsychotic diazepam (Coslop et al. 2022); and, more recently, the antidiabetic metformin (Ferri et al. 2024). For the adsorption of the acetylsalicylic acid and atenolol, a clinoptilolite (SiO_2_/Al_2_O_3_ molar ratio ~ 8.6) from a natural zeolitic tuff was employed, showing an adsorption capacity of 11 and 40 µmol g^−1^ for salicylic acid and atenolol, respectively. These two pharmaceuticals possess electron-donor groups, which favor the interaction with cationic sites in the zeolite. In fact, the atenolol molecule has more atoms that can enter into electron donation processes (two nitrogen atoms and one oxygen), which makes the atenolol adsorption more favorable than for acetylsalicylic acid (Rakić et al. 2013).

The adsorption of the moxifloxacin and norfloxacin was carried out on a natural clinoptilolite under static (batch mode) and dynamic (continuous mode using a packed fixed-ben column) conditions. The adsorption process of the target pharmaceuticals is proposed to occur via cation ex-change plus π-π electron-donor–acceptor interactions between the benzene ring and carboxyl moieties of the antibiotics and the hydroxyl groups on the zeolite surface. The adsorption in the static mode (e.g., 1.7–2.2 mg g^−1^) was higher than in the dynamic one (e.g., 0.5–0.8 mg g^−1^). Generally, the dynamic equilibrium loading is 30–70% of the static capacity. Additionally, a decrease in the dynamic adsorption capacities of both antibiotics is caused by increasing the flow rate of each adsorbate, indicating that the amount of antibiotic adsorbed per mass unit of the zeolite is a function of the contact time of the adsorbate with the adsorbent (Rubashvili et al. 2019).

Another work that tested the adsorption of a pharmaceutical using a column experiment in continuous mode involved a natural Jordanian zeolite for the ibuprofen treatment, achieving removal of up to 78% (Al-rimawi et al. 2019), denoting the high feasibility of this kind of system for practical applications. Such work also evaluated the adsorption of diclofenac, acetaminophen, and indomethacin analgesics but in batch mode. In this last case, it was evidenced by the authors of the work that when the initial concentration of the pharmaceutical increased from 10 to 50 mg L^−1^, the removal capacity also enhanced from 0.04 to 2.0 mg g^−1^. Higher initial concentrations raise the driving force to overcome the mass transfer resistance of pharmaceuticals between the aqueous and solid phases, thus favoring the adsorption on the zeolite.

The pharmaceutical diazepam was treated using a natural clinoptilolite from Brazil (Celta company), as an adsorbent in batch mode, reaching a maximum adsorption capacity of 8.05 mg g^−1^ (Coslop et al. 2022). The authors reported that the adsorption occurred more intensely in the first minutes of treatment, due to the greater availability of sites on the zeolite. The kinetic studies showed that the adsorption of diazepam was a chemisorption process on the surface of the zeolite due to a strong interaction, since the hydrophilic characteristic of the used zeolite.

Another natural zeolitic sample (68.57% clinoptilolite, 17.29% muscovite, 11.66% mordenite, and 2.47% amorphous) from the same Celta company in Brazil was tested for the adsorption of the antidiabetic metformin. The target pharmaceutical was effectively adsorbed by the zeolitic material, and at pH ~ 7.0, the pharmaceutical molecule has a positive charge, driving the electrostatic attraction with the adsorbent surface, which is negative at such pH. Furthermore, it was found that the chemisorption was the rate-limiting step of metformin adsorption. Interestingly, the zeolitic material was regenerated using NaOH solution, achieving ~ 36% of the pharmaceutical desorption in 6 h, and allowing the adsorbent reuse for three consecutive cycles of adsorption/desorption. However, due to the regeneration with the hydroxide, a decrease in the adsorption capacity and collapsing effects on the zeolitic material surface were observed (NaOH induces the disappearance of the clinoptilolite phase) (Ferri et al. 2024).

Apart from zeolites of natural origin, synthetic zeolites have also been widely used for the adsorption of pharmaceuticals from water. For instance, the removals of a fluoroquinolone antibiotic (levofloxacin), a macrolide (erythromycin), and an antiepileptic (carbamazepine) from water using three synthetic organophilic zeolites (Y, mordenite, ZSM-5) were reported by Martucci et al. (2012). Zeolite Y performs better as an adsorbent than mordenite or ZSM-5 zeolites due to the presence of larger pores to host these pharmaceuticals (Martucci et al. 2012). In other work, the adsorption of ketoprofen, hydrochlorothiazide, and atenolol in aqueous solutions on two calcinated Beta zeolites was tested. Such zeolites (which had a synthetic origin with SiO_2_/Al_2_O_3_ ratios of 25 and 360) were obtained by taking their commercial powders from Zeolyst International and carrying out calcination (to remove water and ammonia), raising the temperature from room temperature to 600 °C for 1 h and keeping it at 600 °C for 4 h. The thermal treatment increased the pharmaceutical adsorption efficiency of BEAs regarding the non-calcined. For the calcined Beta25, the saturation capacity increased more significantly. This is due to a higher increase in the acidity of Beta25. Meanwhile, calcined Beta360 was already in acid form.

The adsorption results showed that for these three pollutants, calcined Beta25 was better than calcined Beta360. Since the alumina content is responsible for the hydrophilicity and ionic exchange properties of zeolites, the higher adsorption efficiency of Beta25 concerning Beta360 is related to electrostatic interactions between drug molecules and the zeolite framework. The role of electrostatic interactions with the pharmaceuticals in the adsorption on the zeolites was determined by changing the solution pH (from 2 to 12) (Pasti et al. 2013).

**Fig. 2:**
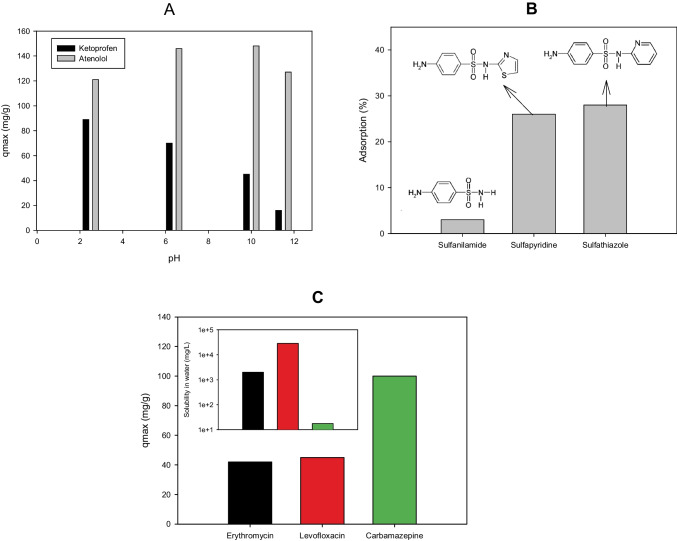
Structural effects on the adsorption of pharmaceuticals on zeolites. **A** pH effect on the adsorption on Beta25 zeolite (figure done by the review authors considering information presented in the reference (Pasti et al. 2013)). **B** Adsorption of structurally related pharmaceuticals on zeolite Y (SiO_2_/Al_2_O_3_ ratio: 200, figure done by the review authors considering information presented in the reference Braschi et al. (2010)). **C** Adsorption of pharmaceuticals having very different structures on zeolite Y (SiO_2_/Al_2_O_3_ ratio: 200, figure done by the review authors considering information presented in the reference Martucci et al. (2012))

The adsorption of ketoprofen on the calcined Beta25 zeolite decreases with the increase of pH due to this pharmaceutical being negatively charged at pH > 4 and experiencing repulsion with the negative charge on the surfaces of beta zeolites (Fig. 2A). Meanwhile, at pH < 4, the driving force for the adsorption of ketoprofen is the hydrophobic interaction of the neutral form of the pharmaceutical. In the case of hydrochlorothiazide, its adsorption displays relative insensitivity up to pH 8 and it decreases gradually for pH > 9 to be practically non-adsorbed at pH 11 (Table 1). Indeed, the undissociated hydrochlorothiazide prevails at pH < 8. Similar to ketoprofen, the decrease in the adsorption with a pH increase can be related to electrostatic repulsions between the zeolite surface and the hydrochlorothiazide anion.

**Table 1:** Adsorption of some pharmaceuticals on natural and synthetic non-modified zeolites

Zeolite	Pharmaceutical	Good adjustment to isotherm model*	Remarkable results on matrix effects	Reference
Clinoptilolite (natural)	Enrofloxacin	Langmuir (at pH: 7 and T: 28 °C)	Adsorption of enrofloxacin is improved by decreasing the pH. Ammonium ions’ presence enhanced the degradation	Ötker and Akmehmet-Balcıoğlu (2005)
		*q*_max_: 19.34 mg g^−1^		
		*K*_L_: 0.17 L mg^−1^		
Natural zeolitic sample (68.57% clinoptilolite, 17.29% muscovite, 11.66% mordenite	Metformin	Langmuir (at T: 25 °C)	Reusability of the zeolitic material for the adsorption of the target pharmaceutical in water	Ferri et al. (2024)
		*q*_max_: 109 mg g^−1^		
		*K*_L_: 0.008 L mg^−1^		
Clinoptilolite (natural)	Diazepam	Langmuir	A cheap and eco-friendly alternative for removing the target pharmaceutical from the water via adsorption. The adsorption of diazepam is a chemisorption process	Coslop et al. (2022)
		*q*_max_: 55.6 mg g^−1^		
		*K*_L_: 0.44 L mg^−1^		
Natural Jordanian zeolite	Paracetamol (acetaminophen)	Langmuir (at T: 25 °C)	Good performance of the zeolite in batch and continuous operation modes for the adsorption of analgesics	Al-rimawi et al. (2019)
		*q*_max_: 8.051 mg g^−1^		
		*K*_L_: 0.344 L mg^−1^		
Y (synthetic)	Levofloxacin	Langmuir	High adsorption percentage (~ 96%) of the pharmaceutical in the outlet of a wastewater plant	Martucci et al. (2012)
		*q*_max_: 45 mg g^−1^		
		*K*_L_: 1.3 L mg^−1^		
Y (synthetic)	Atenolol	Langmuir	The adsorption of the pharmaceutical is higher as the pH is increased from 2 to 10 (pKa: 9.5). The adsorbed pharmaceutical can be released using acetonitrile/formic acid or ammonium acetonitrile/hydroxide mixtures	Sarti et al. (2020)
		*q*_max_: 183 mg g^−1^		
		*K*_L_: 0.11 L mg^−1^		
Y (synthetic)	Sulfachloropyridazine	Langmuir	The adsorption process in water containing dissolved organic matter from forest soil (TOC: 32 g kg^−1^) was quite similar to adsorption in distilled water	Braschi et al. (2010)
		*q*_max_: 280 mg g^−1^		
		*K*_L_: NR L mg^−1^		
ZSM-5 (synthetic)	Carbamazepine	Langmuir	NR	Martucci et al. (2012)
		*q*_max_: 26 mg g^−1^		
		*K*_L_: 0.52 L mg^−1^		
Mordenite (synthetic)	Erythromycin	Langmuir	NR	Martucci et al. (2012)
		*q*_max_: 26 mg g^−1^		
		*K*_L_: 0.07 L mg^−1^		
Beta (synthetic)	Hydrochlorothiazide	Langmuir	Adsorption is approximately constant in the pH range of 2–8, but it decreases gradually for pH > 9 being practically unadsorbed at pH 11	Pasti et al. (2013)
		*q*_max_: 70 mg g^−1^		
		*K*_L_: 0.55 L mg^−1^		

^*^q_*max*_ the saturation capacity, *K*_*L*_ Langmuir coefficient (binding constant), *NR* not reported. Note: The Langmuir isotherm is calculated using the following expression: ${q}_{e}=\frac{{q}_{\text{max}}{K}_{L}{C}_{e}}{1+ {K}_{L}{C}_{e}}$, which is linearized as $\frac{{C}_{e}}{{q}_{e}}=\frac{{C}_{e}}{{q}_{max}}+\frac{1}{{{K}_{L}q}_{\text{max}}}$, where ${C}_{e}$ and ${q}_{e}$ represents the equilibrium concentration of the pharmaceutical and the pharmaceutical amount adsorbed per unit mass of the zeolite, respectively. In turn, ${q}_{\text{max}}$ is the quantity of pharmaceutical adsorbed per unit mass of zeolite required to form the monolayer (also named the saturation capacity). Meanwhile, ${K}_{L}$ is the equilibrium constant of the adsorption process (also called the affinity constant, Langmuir coefficient, or binding constant). Thus, by applying the linear form and a plot of *C*_*e*_/*q*_*e*_ vs. *C*_*e*_, using experimental data, ${q}_{\text{max}}$ can be obtained from the slope and ${K}_{L}$ from the intercept of such a plot

In contrast to ketoprofen and hydrochlorothiazide, the atenolol (which has a basic nature, pKa: 9.5) adsorption on the Beta25 zeolite is increased as the pH changes from 2 to 10, and at pH > 10, the adsorption is decreased (Fig. 2A). This can be explained considering that the pharmaceutical is protonated at pH < 9, whereas in basic solution, the neutral molecule is the dominant species. Therefore, for atenolol, electrostatic interactions with the zeolite can take place at pH < 9, while for the neutral molecule (pH > 10), the adsorption is caused by hydrophobic interactions, and such molecules are preferentially adsorbed onto the hydrophobic framework of the zeolite (Pasti et al. 2013).

Sarti et al. also evaluated the adsorption of pharmaceuticals by comparing the SiO_2_/Al_2_O_3_ ratio. They tested two synthetic Y zeolites, with SiO_2_/Al_2_O_3_ ratios of 30 and 200, as sorbents for ketoprofen, hydrochlorothiazide, and atenolol in an aqueous matrix (Sarti et al. 2020). Adsorption of ketoprofen and hydrochlorothiazide on Y200 is better than that on Y30, because of the higher hydrophobic nature of the zeolite Y200 (due to a higher SiO_2_/Al_2_O_3_ ratio), and Y200 also has a superior penetration capability of the target molecules inside its structural microporosity. On the contrary, the adsorption of atenolol on Y30 is more favored. This last finding is ascribed to electrostatic interactions between Y30 (which has more negative charges than Y200) and atenolol which is positively charged at the experimental conditions tested by the authors (Sarti et al. 2020).

Braschi et al. (2013) tested the adsorption of six sulfonamide antibiotics (i.e., sulfanilamide, sulfapyridine, sulfathiazole, sulfadimethoxine, sulfadoxine, and sulfamerazine) onto a synthetic high silica zeolite Y (HSZ-Y, SiO_2_/Al_2_O_3_ ratio: 200) with hydrophobic properties (Braschi et al. 2013). Among those sulfonamides, sulfanilamide showed the lowest adsorption on HSZ-Y, which was ascribed to its high hydrophilicity (sulfanilamide has the presence of a hydrogen atom at the sulfonamide group, in the place of a heterocyclic aromatic ring as the other sulfonamides, as exemplified in Fig. 2B). For the other antibiotics, it was found the embedding of sulfa drug molecules inside zeolite cages, in addition to the loading of sulfa drugs on the external zeolite surface. Furthermore, the authors proposed the formation of dimeric species (through intermolecular hydrogen bonding of the nature N–H…O and N–H…N) inside zeolite cavities, especially for those sulfonamides of the lowest dimensions such as sulfathiazole and sulfapyridine (Braschi et al. 2013). In turn, the comparison of adsorption of sulfamethoxazole (at ~ 29 µmol L^−1^) into three synthetic high silica zeolites (Y, mordenite, and ZSM-5 having SiO_2_/Al_2_O_3_ ratios of 200, 200, and ~ 500, respectively) showed that after less than 1 min, ~ 75% of this sulfonamide was adsorbed on zeolite Y and MOR (Blasioli et al. 2014). Meanwhile, adsorption equilibrium for sulfamethoxazole was not achieved even after 2 weeks on ZSM-5. Among the three tested zeolites by Blasioli et al., ZSM-5 has the smallest channel system and the reduced diffusivity of drug molecules into its pore system, which limits the sulfamethoxazole adsorption (Blasioli et al. 2014).

In another work, sulfonamides have also been adsorbed on a synthetic faujasite zeolite (a highly dealuminated Y-type, SiO_2_/Al_2_O_3_ ratio: 200) (Braschi et al. 2010). More than 90% of sulfadiazine, sulfamethazine, and sulfachloropyridazine (at 40 µmol L^−1^ of initial concentration each) were removed from water by this zeolite at a very short treatment time (< 1 min). Despite the fast and favorable adsorption of the sulfonamides, detailed kinetics were not considered by the authors of the original research, and this could be an interesting topic to develop in future works. Also, it is important to mention that the large cages and windows on the faujasite zeolite (Y) make this structure useful for the adsorption of sulfonamide antibiotics, and a hydrophobic-type adsorption mechanism is proposed. The adsorption was associated with interactions with zeolite pores of different dimensions, being higher in micropores and lower in larger pores (Braschi et al. 2010). However, it is important to mention that the water solubility of the organic pollutant is also a useful parameter in explaining differences in the adsorption of structurally related compounds on the same high-silica zeolite. For instance, in the comparison of adsorption of erythromycin, levofloxacin, and carbamazepine on a synthetic zeolite Y (Si/Al: 200), the most hydrophobic pharmaceutical (i.e., the least soluble in water, carbamazepine in this case) is more efficiently adsorbed than the hydrophilic ones (Fig. 2C) (Martucci et al. 2012).

**Fig. 3:**
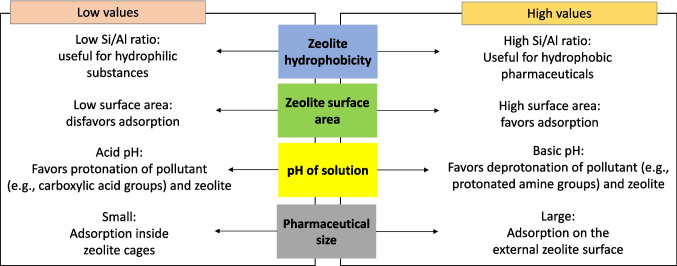
Scheme of the effects of relevant parameters at low and high values that influence the adsorption of pharmaceuticals in water on non-modified zeolites

On the other hand, considering the reviewed works about the adsorption of some pharmaceuticals using natural and synthetic zeolites, Table 1 was made. It can be noted that the adsorption of diverse pharmaceuticals on zeolites adjusts well to the Langmuir isotherm model (see the footnote of Table 1), which assumes that adsorption is a homogenous process in which the adsorbate (i.e., pollutant) is adsorbed in a monolayer (the thickness of the layer is of the order of one molecule) onto the adsorbent, taking place at a fixed number of adsorption sites with no lateral interaction or transmigration (Waheed et al. 2021). Additionally, few works explore the adsorption in complex matrices (more research on this topic could be developed in the future). However, those works that evaluate such a topic report good performances (i.e., high removal percentages of pharmaceutical adsorption even in complex matrices such as the effluent of a wastewater treatment plant). Also, it can be highlighted that some other works deal with the pH effect, evidencing that this parameter alters the adsorption of pharmaceuticals on zeolites (Table 1). In addition to the information shown in Table 1, a scheme is elaborated and presented in Fig. 3. This figure summarizes four relevant aspects (hydrophobicity, surface area, pH, and pollutant size) linked to the adsorption of pharmaceuticals on non-modified zeolites, which were presented above.

### Adsorption of pharmaceuticals from water using modified zeolites

**Fig. 4:**
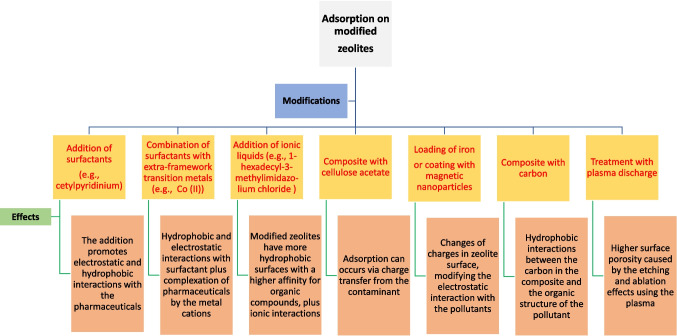
Zeolites modifications to enhance the adsorption of pharmaceuticals

The modification of natural and synthetic zeolites is an alternative to favor the adsorption of pharmaceuticals. Different strategies such as the addition of surfactants, transition metals, ionic liquid, modification with HCl, loading of iron or coting with nanoparticles, and the formation of composites have been used (Fig. 4). For instance, a natural zeolite (raw clinoptilolite-rich zeolitic tuff) was modified with cetylpyridinium chloride (a cationic surfactant) to absorb diclofenac from aqueous samples. The zeolite modification involved the surfactant in amounts equivalent to 100, 200, and 300% of its external cation-exchange capacity (ECEC, which is a measure of how many cations can be retained on the external surface of the zeolite, and it is determined by the method of Ming and Dixon (Ming and Dixon 1987)). A monolayer of cetylpyridinium occurred at the zeolitic surface by loading of the surfactant level equal to its external cation-exchange capacity, whereas higher amounts of the surfactant led to less extended bilayers, ordered bilayers, or admicelles at the surface of the zeolite (Krajišnik et al. 2011; Roshanfekr Rad and Anbia 2021).

The non-modified zeolite (which has a net negative charge and inorganic cations at the hydrophilic surface) had no affinity for either hydrophobic or anionic species (such as diclofenac at pH 7.4). In turn, the zeolite modified with the surfactant showed a high affinity toward the pharmaceutical, and the pollutant adsorption increased as the surfactant uploading was higher. For the modified zeolites, the bilayer of surfactant at the zeolitic surface results in charge reversal on the external surface, providing sites where anion substances are retained. At high cetylpyridinium concentrations, in addition to the interactions between the positively charged surface of the modified zeolite and the anionic form of diclofenac, hydrophobic interactions between the alkyl chains of the surfactant and hydrophobic parts of the pharmaceutical are possible (Krajišnik et al. 2011).

In other studies, Krajišnik et al. also tested the adsorption of diclofenac sodium (and ibuprofen) but this time using the clinoptilolite zeolite modified with hexadecyltrimethylammonium bromide or benzalkonium chloride (cationic surfactants). They reported the modification of the zeolitic surface in two levels (equal to and twice the external cation-exchange capacity of the zeolitic tuff). It was informed that a proportional increase in the adsorption of pharmaceuticals by increasing the amount of surfactant used for modification. Also, it is proposed that the hydrophobic and electrostatic interactions between the pharmaceutical and the surfactants on the clinoptilolite surface favor the uptake of the pollutants (Krajišnik et al. 2010, 2013).

Clinoptilolite and phillipsite-rich tuffs (two natural sources, which had 79 and 58% of zeolitic content, and Si/Al ratios of 4.89 and 2.45 for clinoptilolite and phillipsite, respectively) were modified with cetylpyridinium chloride (one-tail cationic surfactant) or Arquad® 2HT-75 (two-tail cationic surfactant). The modification is done using a fast functionalization method, involving aqueous suspension of proper surfactant amounts and initial zeolitic tuff, allowing the surfactant adsorption by mixing at 5000 rpm and 50 °C for 15 min; with an ulterior filtration step using ashless filter paper for obtaining the solid modified zeolitic material. The modified zeolitic materials have been compared for the removal of ketoprofen and diclofenac from water. The surfactants can form mono- or bilayers. For both zeolitic materials and surfactants, the best adsorption results were obtained for the bilayer form. The adsorption of pharmaceuticals involves ionic and hydrophobic mechanisms, being the ionic exchange of the chloride counter ion dominant as both drugs were anionic forms, and the positively charged bilayers favor the pharmaceuticals adsorption (Smiljanić et al. 2020). Analogous results have been found for the adsorption of salicylic acid on the same family of natural zeolitic materials (clinoptilolite and phillipsite) modified with cationic surfactants (Smiljanić et al. 2021a).

The adsorptions with the zeolites modified with the one-tail surfactant were higher than those obtained for the modification with the two tails, indicating that the surfactant structure has a strong influence on the adsorption of pharmaceuticals. Besides, the modified zeolites showed higher removals for diclofenac than ketoprofen, which was related to the hydrophobicity of the target molecules. As diclofenac is more hydrophobic (Log *K*_ow_: 4.52) than ketoprofen (Log *K*_ow_: 3.17), the former has higher hydrophobic interactions with the surfactants, favoring its adsorption (Smiljanić et al. 2020; Roshanfekr Rad and Anbia 2021).

Smiljanić et al. (2021b) also tested clinoptilolite and phillipsite modified with cetylpyridinium chloride and Arquad® 2HT-75 surfactants to remove ibuprofen and naproxen from aqueous media. Analogous results to those reported above were found. The findings indicate that the adsorption of pharmaceuticals exhibited both ionic and hydrophobic mechanisms, dominating the ionic exchange. The phillipsite zeolite modified with bilayers of the two-tail surfactant (i.e., Arquad® 2HT-75) had a slightly lower adsorption capacity. Probably the two alkyl tails prevent a closer packing of the surfactant molecules at the phillipsite surface, and this could reduce either partitioning or anion exchange for the adsorption of the pharmaceuticals. Additionally, the modified zeolites showed preferential adsorption toward ibuprofen (Log *K*_ow_: 3.97) due to its higher hydrophobicity than naproxen (Log *K*_ow_: 3.18) (Smiljanić et al. 2021b; Roshanfekr Rad and Anbia 2021).

On the other hand, the modification of a clinoptilolite-rich rock with one-tail cationic surfactants, via replacement of the exchangeable cations by long-chain species (cetylpyridinium chloride, benzalkonium chloride, and hexadecyltrimethylammonium chloride or bromide) and forming patchy or complete bilayer, to adsorb ibuprofen (in its anionic form) has been informed. The ibuprofen adsorption was a function of the type of counterion and morphology of the surfactant. The zeolites modified using chlorinated surfactants have a slightly higher value of the Cl^−^ hydrated radius compared to that of Br^−^, making chlorinated surfactants unable to form a complete bilayer onto the surface of the clinoptilolite. Also, the ring-containing surfactant (i.e., benzalkonium chloride) limits the bilayer formation. Consequently, the adsorption of ibuprofen using the zeolites modified with chloride-surfactants occurs by a combination of external anionic exchange and partition into the hydrophobic part of the patchy bilayer (Izzo et al. 2019).

The combination of surfactant cations with extra-framework transition metals is another possible way to improve the adsorption of pharmaceuticals such as salicylic and carbamazepine on zeolites. A synthetic zeolite (NaY, Si/Al ~ 2.8) was modified through the incorporation of Co^2+^, Ni^2+^, or Cu^2+^, and the dual combination of these metal cations with cetylpyridinium (CPY^+^). The modified zeolites had a higher uptake of salicylic acid and carbamazepine than the NaY (Cabrera-Lafaurie et al. 2014).

For zeolites modified with metals and CPY^+^, the total surface area and pore volume are decreased (~ 30–65% lower) regarding NaY, which is attributed to the larger amounts and size of the surfactant ions (a portion of these species probably occupies space in the supercages of the zeolite framework). Then, the adsorption of salicylic acid and carbamazepine onto the modified zeolites is controlled by a combination of pharmaceutical-surfactant hydrophobic interactions, with electrostatic interactions, and the complexation of the pharmaceuticals by the metal cations (Co^2+^, Ni^2+^, or Cu^2+^) through the aromatic rings, N-, and/or O-donor moieties on the organic pollutants (Cabrera-Lafaurie et al. 2014).

Modifications of natural zeolites using only cations (without surfactants) of transition metals have also been informed. As an example, a zeolitic tuff (72.6% clinoptilolite phase) modified with cations of Mn, Ni, Cu, and Zn (which synthesis firstly converted the zeolitic material into the Na-rich form, followed by an ion exchange using the chloride salts of the transition metals) was used to adsorb pharmaceuticals. Salicylic acid was adsorbed on the modified materials. All the modified minerals presented improved adsorption capacities toward salicylic acid compared to the natural one, which is associated with the formation of complexes of the metal ions with the pharmaceuticals. However, the adsorption on the minerals modified with Cu and Zn exhibited the saturation of these solids, while the adsorption on those modified with Ni and Mn pointed to a multilayer system, thus remarking a strong influence of the metal ion (Rakić et al. 2013).

Natural zeolite modifications with acid to favor the adsorption of pharmaceuticals are also plausible. Rubashvili et al. (2019) modified clinoptilolite with HCl (the natural mineral was treated with an acid solution at 400 °C) and evaluated it to adsorb moxifloxacin and norfloxacin from water. The modification of the zeolite with acid enhanced the removal of the considered antibiotics, which is connected to changes in the pore sizes that provide a large surface area for the adsorption process (Rubashvili et al. 2019).

The modification of zeolites can also involve the addition of ionic liquids (IL) to enhance the adsorption capability. For instance, the removal of chloramphenicol in water using a natural zeolite (obtained from the St. Cloud Mine in Winston, New Mexico) modified by 1-hexadecyl-3-methylimidazolium chloride (i.e., the IL) has been investigated (Sun et al. 2017). The modification of such a zeolite by the IL at 50, 100, and 200% of the external cation exchange capacity induced an increase in the adsorption of chloramphenicol, which is evidenced by the increase in the distribution coefficient (*K*_d_: concentration in the zeolite/concentration in the liquid), from 2.3 to 4.5 L kg^−1^ as the IL amount is augmented.

The IL-modified zeolites show higher contact angles with water than the raw material, indicating that the modified zeolites have more hydrophobic surfaces with a higher affinity for organic compounds such as chloramphenicol. Indeed, the adsorption mechanism is associated with hydrophobic interactions between the pharmaceutical and the non-polar tail of the IL on the modified zeolites (external sites), plus the electrostatic interactions of the positive charges of the mineral surface with the partially negatively charged functional groups of chloramphenicol (external sites) (Sun et al. 2017).

On the other hand, zeolites (such as LTA-type, e.g., zeolite A) can be composited with cellulose acetate to produce a reusable adsorbent fiber (named ZCA). This kind of blended material has been used to remove the antibiotic erythromycin from aqueous samples. The zeolites are embedded in the cellulose acetate matrix. The composite material exhibited higher adsorption properties and reusability than the zeolite or cellulose acetate fiber individually. The adsorption of the antibiotic on ZCA fitted well to a pseudo-second-order kinetic model and had a *q*_max_ of 358 mg g^−1^. Additionally, quantum chemical analyses suggested that the adsorption occurs via charge transfer from erythromycin to ZCA (Jodeh et al. 2021).

A natural zeolite (clinoptilolite) loaded with ferrous ions has been used for the adsorption of tartrazine (one of the rawest materials in the food and pharmaceutical industries), showing that the adsorption can be well fitted with the Langmuir model with *q*_max_: 1.30 mg g^−1^ (Russo et al. 2021). However, the adsorption performance of the modified zeolite was not compared to the raw one. Besides iron loading, a different way to modify the adsorptive properties of zeolites comprises the coating with magnetic nanoparticles. Salem Attia et al. (2013) reported the synthesis and application of a zeolite coated by iron oxide nanoparticles (named MNCZ, which is obtained by mixing at pH 10 a sample of natrolite zeolite with ferrous sulfate and ferric chloride using ultrasonication, followed by a stirring in a rotary shaker at 160 rpm for 24 h, plus the treatment with concentrated sodium hydroxide to form the target precipitate; thus, black particles of size ~ 10–20 nm are generated). The MNCZ was employed for removing diverse pharmaceuticals (diclofenac, naproxen, gemfibrozil, and ibuprofen) from water.

The modified zeolite by nanoparticle coating (i.e., MNCZ) had a high adsorption potential for the target studied pharmaceuticals, and the removal efficiency was more than 95% within only 10 min (but no comparison with the non-modified zeolite was presented; thus, the role of the coating is not clearly understood). The authors of that work reported a pseudo-second-order model as the best-fit model with the experimental data. This is a very useful model when the initial concentration of the solute is not high. Such a model relies on the assumption that chemisorption may be the rate-limiting step of the adsorption. Besides, for the system using MNCZ, as the solution pH was changed from 2 to 11, the adsorption of the pharmaceuticals decreased. Lower sorption efficiency at basic pH values is attributed to the increase in negative charges at the modified zeolite surface, affecting the interaction with the considered pollutants, which are acidic compounds having pKa < 5 (e.g., carboxylic acids). Furthermore, the adsorption of diclofenac is the most favored, because this analgesic contains N–H and O–H moieties, which allows it more interaction with the modified zeolite than the other three pharmaceuticals (where O–H is the dominant functional group) (Salem Attia et al. 2013).

More recently, a nanosized zeolite composited with carbon has been used for the adsorption of the antibiotic ciprofloxacin (Al-jubouri et al. 2022). This composite was synthesized using a ZSM-5 zeolite. For the composite preparation, ZSM-5 zeolite nanocrystals are dispersed in water, and it is mixed with sucrose for 10 min over a hot plate at 100 °C. Then, the carbon is mixed with the zeolite-sucrose mixture for 15 min. After that, citric acid is mixed with the above mixture for 5 min, producing a slurry that is heated at 100–110 °C with continuous stirring, and the resultant solid particles are carbonized at 600 °C for 2 h in an air-free atmosphere to obtain the composite.

The above-presented composite has a higher surface area than the carbon support or the nanosized zeolite, which favors the sorption of the pharmaceutical on the composite. Moreover, ciprofloxacin removal by the composite is dependent on the pH due to the adsorbent surface charges and acidic/basic functional groups of the antibiotic. In fact, the highest adsorption of ciprofloxacin on the nanosized zeolite composited with carbon (which has a point of zero charge, PZC: 8.1) occurs at pH between 4 and 6. In turn, when the solution pH is above 8, the ciprofloxacin molecule loses the proton from the carboxyl group being an anion. Then, the repulsion forces between the composite particles and the antibiotic are increased because both have negative charges, thus resulting in low removals of the pollutant from water.

A composite of synthetic zeolite and carbon has also been applied to adsorb erythromycin from water. Compared to the zeolite alone, the composite shows a higher maximum adsorption capacity and very fast removal of the pharmaceutical from real wastewater. The hydrophobic interactions between the carbon component in the composite and the organic structure of the pollutant (which is rich in methyl and methylene groups, providing a hydrophobic nature) governed the adsorption process (Grela et al. 2023). Another recent work also presents the utilization of similar zeolite-carbon composites for the removal of colistin, fluoxetine, amoxicillin, and 17-alpha-ethinylestradiol in aqueous samples. This last work showed that due to the hydrophobic nature of the composite, fluoxetine and 17-alpha-ethinylestradiol (the most hydrophobic pollutants) are removed more efficiently, and such removal is mainly associated with a physical sorption. Moreover, the regeneration of the material by washing using methanol demonstrated that the composite is reusable, exhibiting fluoxetine adsorption efficiencies higher than 80% even after the fifth regeneration cycle (Bajda et al. 2024).

Apart from the addition of reagents or composite formations, zeolites can be modified by the treatment with plasma discharge. Previous works about the sorption of diclofenac on plasma-treated clinoptilolite showed an increase in the adsorption capacity from 52 to 64% using the plasma-treated vs. the untreated zeolite. The adsorption improvement is associated with a higher surface porosity caused by the etching and ablation effects of the impinging energetic particles from the plasma discharge. The authors proposed as a possible mechanism of adsorption of the pharmaceutical, the electrostatic attraction between the surface of the zeolite and diclofenac through cation exchange (Garcia et al. 2019).

## Degradation of pharmaceuticals using zeolites in AOTs

AOTs produce in situ short-life species that have high redox potentials such as hydroxyl radicals (HO•, E°: 1.90–2.70 V) or sulfate radicals (SO_4_• − , E°: 2.60–3.10 V) (Lee et al. 2020); that is, these radicals are very strong oxidizing agents. AOTs are recognized for their robust degrading capability toward organic contaminants. Among the AOTs, catalytic processes are gaining attention from the scientific community because of their elevated possibility to generate radical species, high efficiency for the removal of pollutants, and good operational results under diverse experimental conditions (Kohantorabi et al. 2021; Araújo et al. 2021; Issaka et al. 2022). On the other hand, zeolites are low-cost materials that have been successfully involved in AOTs such as catalytic ozonation, Fenton-based systems, and activation of persulfates, which are detailed in the following subsections.

### Degradation and transformation of pharmaceuticals by catalytic ozonation involving zeolites

Ozone (O_3_) alone can attack and induce oxidations on some pharmaceuticals in water samples. However, O_3_ is a selective oxidant (i.e., this reacts with some specific functional groups in the structure of the contaminants). Besides, the adsorption on the zeolites is a phase change of the pharmaceuticals (from liquid to solid phase), and after the adsorption, the zeolite is saturated/polluted with pharmaceuticals. Therefore, the combination of zeolites with O_3_ is a good option to degrade the adsorbed pharmaceuticals and regenerate the solid material (Eqs. 1 and 2).

1 $$Zeolite+pollutant\to Zeolite-{pollutant}_{(adsorbed)}$$

2 $$Zeolite-{pollutant}_{(adsorbed)}+{O}_{3}\to {Zeolite}_{(regenerated)}+degradation\;product$$

The zeolite/O_3_ interaction can also change the pore structure in the zeolite (Ötker and Akmehmet-Balcıoğlu 2005). It should be mentioned that zeolites can contain ions of metals (e.g., compensation cations, modifications) or be decorated with metal oxides, and these metal-based species can react with ozone, producing radicals. In contact with water, these oxides on the zeolite surface (which is represented by S) will be covered with hydroxyl groups (S-OH), which behave as Brønsted acid sites (Valdés et al. 2009), thus forming S-OH_2_^+^ that promote the generation of radicals (Eqs. 3 and 4). Moreover, some metal cations (Lewis’s acids, e.g., Fe^2+^ or Co^2+^) in the zeolites can also act as catalytic active sites, decomposing ozone into radicals (Eqs. 5 and 6) (Issaka et al. 2022).

3 $$S-{{OH}_{2}}^{+}+{O}_{3}\to S-{OH\cdot }^{+}+{HO}_{3}\cdot$$

4 $${HO}_{3}\cdot \to HO\cdot +{O}_{2}$$

5 $${Fe}^{2+}+{O}_{3}+{H}_{2}O\to {Fe}^{3+}-OH+HO\cdot + {O}_{2}$$

6 $${Co}^{2+}+{O}_{3}+{H}_{2}O\to {Co}^{3+}-OH\cdot +{O}_{2}$$

Chemical oxidation through ozone in the presence of zeolite-based catalysts is an attractive option for degrading refractory pollutants such as pharmaceuticals because the zeolite/O_3_ combination accelerates the degradation of pollutants and promotes the zeolite cleaning (Valdés et al. 2009). This is the case of enrofloxacin adsorbed on a natural zeolite (clinoptilolite), which was treated with ozone for 30 min, leading to both decontamination/cleaning of the used zeolite and degradation of enrofloxacin toward biodegradable products (Ötker and Akmehmet-Balcıoğlu 2005).

Ibuprofen has also been treated by both non-catalytic and catalytic ozonation. Around 93% of ibuprofen was degraded after 4 h of ozonation under optimal conditions, and the catalytic ozonation using H-Beta (acidic) or Fe-H-Beta (iron-modified) zeolites showed a significant improvement in the degradation rate of this analgesic; degradation rate increases with increasing catalyst amount because more catalytic sites are available to promote the activation of ozone for the degradation of pollutants. Moreover, the iron-modified catalyst exhibited a higher degradation of ibuprofen compared to the acidic zeolite (Saeid et al. 2018). The authors did not provide explanations for these results. However, it could be suggested that the Brønsted acid sites in the zeolite promote the formation of radicals (Eqs. 3–4), accelerating pollutant degradation regarding ozonation alone. The increase in the zeolite concentration provides more acid sites, and the modification of the zeolite with iron makes easier the formation of HO• (Eq. 5), thus improving the elimination of ibuprofen (Valdés et al. 2009).

It is important to mention that ibuprofen can be degraded by the action of ozone alone, inducing an oxidation of alkyl moiety toward a ketone group (Table 2). However, some intermediate products are more resistant to O_3_ than the parent compound, requiring the catalytic ozonation with zeolites to improve the mineralization of the pollutant. Thereby, ibuprofen was treated by H-Beta zeolites modified with Cu or Ni. These zeolites increase the elimination kinetics of the intermediate products, being the best results when using Cu-H-Beta catalysts. As the Cu-H-Beta zeolite is a hydrophobic material, this can induce the adsorption of ibuprofen from water and favor the degradation by catalytic ozonation (Saeid et al. 2020a).

**Table 2:**
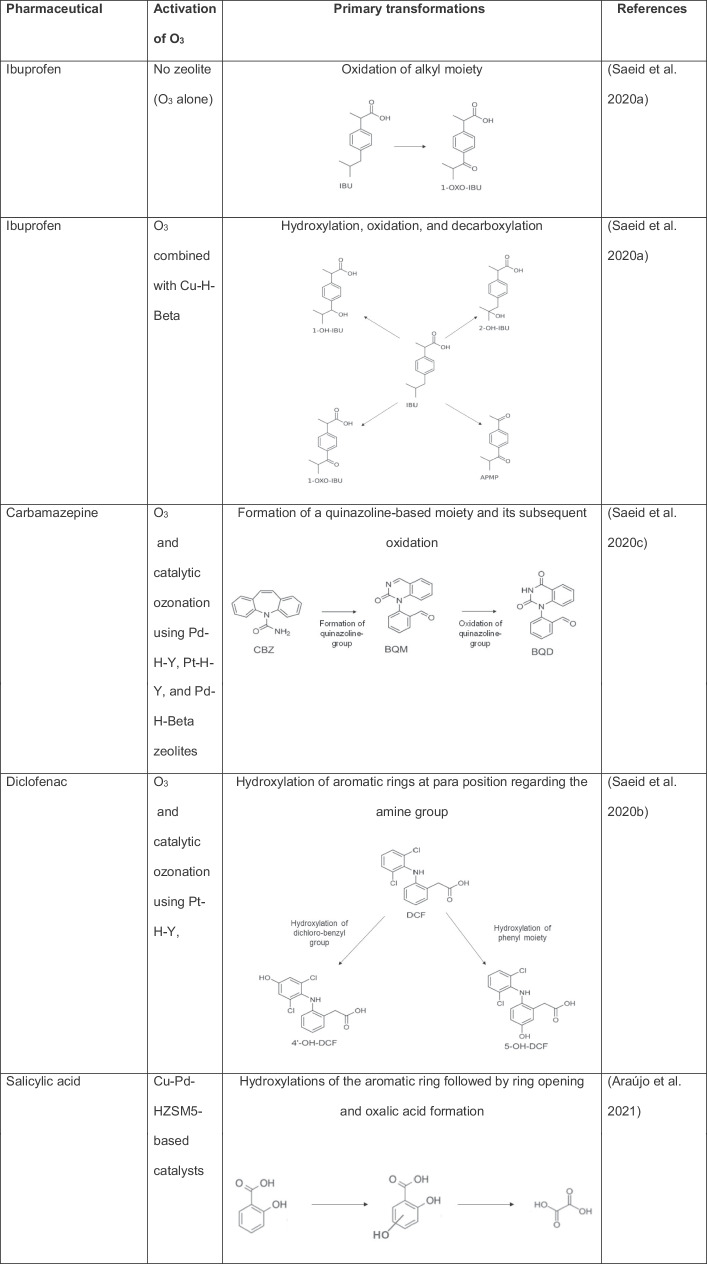
Main primary transformations of pharmaceuticals by ozonation and catalytic ozonation using zeolites*

^*^Abbreviations: IBU: ibuprofen, *1-OXO:* 1-oxo-ibuprofen, *1-OH-IBU:* 1-hidroxy-ibuprofen, *2-OH-IBU*: 2-hidroxy-ibuprofen, *APMP:* 1-(4-acetylphenyl)-2-methylpropan-1-one, *CBZ:* carbamazepine, *BQM*: 1-(2-benzaldehyde)-4-hydro-(1H,3H)-quinazoline-2-one, *BQD:* 1-(2-benzaldehyde)-(1H,3H)-quinazoline-2,4-dione, *DFC:* diclofenac, 4′-OH-DFC: 4′-hydroxy-diclofenac, *5-OH-DFC:* 5-hydroxy-diclofenac

The modification of the catalyst with copper is important in the degradation of the pharmaceutical because it provides catalytic active Cu sites and the presence of acid sites (both types of sites are dependent on the amount of Cu, Cu particle size, and Cu dispersion on the zeolite). We should mention that the primary ibuprofen transformation by catalytic ozonation using Cu-H-Beta zeolite occurs via hydroxylation, oxidation, and decarboxylation mainly (Table 2). Subsequently, the aromatic rings open to form small organic acid molecules, and then carbon dioxide and water, wherein the intermediates are generated and degraded at a higher speed than in classical ozonation (Saeid et al. 2020a).

Carbamazepine and diclofenac are other pharmaceuticals degraded by catalytic ozonation with zeolites (Saeid et al. 2020c, b). Similar to the case of ibuprofen, carbamazepine and diclofenac are quickly degraded by O_3_ alone (which typically modifies/attacks the central double bond of carbamazepine and the aromatic rings on diclofenac structures, respectively, Table 2), generating by-product resistant to ozonation. For the treatment of carbamazepine, Pd-H-Y, Pt–H-Y, and Pd-H-Beta zeolites were used as catalysts to improve the ozonation performance, exhibiting low leaching of the noble metals. The Pd-H-Y material shows better results for the degradation of the carbamazepine intermediates thanks to the highest Brønsted acidity and moderately high Lewis-type acidity that facilitate the formation of degrading radicals. Additionally, the process performance is enhanced when the temperature of the system is increased from 20 to 50 °C. At those temperatures, dissolved oxygen is adsorbed on metal oxides of the zeolite, producing active atomic oxygen species, as well as lattice oxygen atoms. Such species are useful for oxidizing organic molecules (Saeid et al. 2020c).

The degradation of diclofenac using the Pt–H-Y in combination with O_3_ led to faster degradation of the pharmaceutical and its by-products compared with the sole ozonation action. The mechanisms that enhance the degradation by the presence of the zeolite may consist of an O_3_ adsorption on the catalyst surface, followed by the generation of active radical species that attack the organic substances in the solution, or adsorbed diclofenac and by-products on the zeolite surface, which react with the ozone dissolved in the solution. Also, another pathway that is the interaction of O_3_, diclofenac, and its intermediates (all three species adsorbed on the catalyst surface) can contribute to the mineralization (Saeid et al. 2020b). The catalytic ozonation primarily transforms diclofenac into hydroxylated by-products from the attack to its aromatic rings, as illustrated in Table 2.

Mono- (Pd or Cu) and bimetallic (Pd-Cu or Cu-Pd) zeolitic structures (ZSM-5 type) have been used to deal with salicylic acid by catalytic ozonation. Metal-zeolite catalysts positively influenced the degradation of the pharmaceutical regarding the zeolites non-modified with the metals (Araújo et al. 2021). The catalytic activity of those zeolites is determined by a combination of the acidic properties and the chemical composition at the surface, following the performance order: CuPd-HZSM5 > PdCu-HZSM5 ≃ Pd-HZSM5 > Cu-HZSM5 > HZSM5. The HZSM5-based catalysts had high hydrophobicity, which can favor the initial adsorption of the salicylic acid molecules on the catalyst and their subsequent degradation. Furthermore, the excellent catalytic activity of the metal-containing HZSM5 materials is also attributed to the presence of different concentrations and oxidation states of the metal species. Especially, when M(0)/M(II) ratios are higher, better degradation results are observed.

The HZSM5-based catalyst series lead to primary transformations of salicylic acid via initial hydroxylations of the aromatic ring followed by ring opening and oxalic acid formation (Table 2), in addition to high mineralization (> 80%), even after the fifth reuse cycle for the CuPd-HZSM5 zeolite. Indeed, leaching of Pd or Cu and morphology changes are not found at the end of the degradation tests, indicating the high stability of this zeolite in the process (Araújo et al. 2021). It is important to remark that in catalytic ozonation, the catalysts improve the decomposition of molecular ozone to generate highly active free radicals or favor the interaction between O_3_ and the target pollutant or its intermediates, thus facilitating the mineralization of organic matter (Issaka et al. 2022).

To enhance the degrading performance, catalytic ozonation has been combined/merged with other water treatment processes. Recently, a hybrid system involving electro-flocculation and catalytic ozonation (using a novel catalyst Ni-Co-Zeolite 5A), followed by ceramic membrane filtration, has been applied for treating pharmaceutical wastewater containing the antibiotics tylosin tartrate and enrofloxacin (Masood et al. 2023). Before the merging of the processes, the optimization of operational parameters of the individual systems shows that the increase of the cell potential in the electro-flocculation from 5 to 15 V enhanced significantly the removal of the target pharmaceuticals, the chemical oxygen demand (COD), and turbidity of the pharmaceutical wastewater sample. Similarly, the increase of zeolite dose from 5 to 15 mg L^−1^ in the catalytic ozonation augmented the process efficiency, even being higher than only ozonation. The combined treatment was more efficient than the single systems, leading to higher elimination of the targeted contaminants and superior removals of COD and turbidity in a shorter treatment time. The authors propose that in the electro-flocculation, the aluminum and iron electrodes produce ions in the bulk that interact and form an abundant number of hydroxides (e.g., Fe(OH)_3_ and Al (OH)_3_) as sweep flocs during electrochemical reactions that adsorbed the pollutants from water. Meanwhile, in the combination of processes, degradation improvement could be associated with an enhancement in the production of HO• by the catalytic ozonation through O_2_ reduction to form H_2_O_2_ that further elevates the transformation of O_3_ into radicals, where Ni and Cu metals on the zeolite can play a relevant role in redox steps involving oxygen and ozone. Besides, the incorporation of the ceramic membrane, after the combination of electro-flocculation and catalytic ozonation, augmented the removal of pollution (turbidity, COD, and antibiotics) by acting as a filtration physical barrier (Masood et al. 2023). Interestingly, the reusability of the zeolitic catalyst and ceramic membrane was very high. In the hybrid treatment, the removal efficiency of pollutants after the third repeated run was retained due to the continuous regeneration of the catalyst surface and membrane fouling mitigation. The ozone action, electrochemical oxidation, and the attacks of the produced radicals lead to the degradation of pollutants, which avoids the poisoning of the catalyst and also increases the membrane filtration life.

### Zeolites in Fenton-based processes

The Fenton-based processes involving zeolites also lead to the generation of hydroxyl radicals through the interaction of hydrogen peroxide with diverse iron species incorporated into the zeolites. For instance, iron-exchanged zeolites have been used for organic pollutant degradation through the reaction between the ferric ions in the zeolite and H_2_O_2_ in the presence of UV light (Eqs. 7–10) (Kušić et al. 2006; Perisic et al. 2016). Zeolites can support iron nanoparticles, where Fenton oxidation is generally attributed to two heterogeneous catalysis mechanisms (Eqs. 11 and 12) and the induced homogeneous Fenton oxidation (Eqs. 7–8) by the leaching of iron (Anis and Haydar 2019). Also, zeolites containing intraframework iron oxyhydroxides (FeOOH) have shown a catalytic activity to decompose H_2_O_2_ for organic pollutant elimination (Shen et al. 2016). Some of the iron oxides supported on zeolites have semiconductor properties; their interaction with UV light generates electron–hole pairs (Eq. 13), and the electrons can promote the H_2_O_2_ reduction to form hydroxyl radicals (Eq. 14) useful for contaminants degradation (Takdastan et al. 2020).

7 $${Fe}^{2+}+{H}_{2}{O}_{2}\to {Fe}^{3+}+HO\cdot +{OH}^{-}$$

8 $${Fe}^{3+}+{H}_{2}{O}_{2}\to {Fe}^{2+}+HOO\cdot +{H}^{+}$$

9 $${Fe}^{3+}+{H}_{2}O+{UV}_{light}\to {Fe}^{2+}+HO\cdot + {H}^{+}$$

10 $${H}_{2}{O}_{2}+{UV}_{light}\to 2 HO\cdot$$

11 $$={Fe}^{2+}+{H}_{2}{O}_{2}\to ={Fe}^{3+}+HO\cdot +{OH}^{-}$$

12 $$={Fe}^{3+}+{H}_{2}{O}_{2}\to ={Fe}^{2+}+HOO\cdot +{H}^{+}$$

13 $$FeyOx@zeolite+{UV}_{light}\to FeyOx@zeolite(h+/e-)$$

14 $${e}^{-}+{H}_{2}{O}_{2}\to HO\cdot +{HO}^{-}$$

Zeolites such as Fe-exchanged ZSM-5 (Fe-ZSM-5) have been involved in Fenton-based processes to eliminate phenolic structures (which are typical moieties present in pharmaceuticals). The efficiency of the catalytic process is highly dependent on the concentration of iron ions. A higher dosage of Fe-ZSM5 provides a higher overall amount of iron ions in the zeolite framework, as well as leached iron ions into the solution bulk. Both iron forms (leached and in the zeolite framework) induce the generation of hydroxyl radicals from the hydrogen peroxide decomposition, resulting in the degradation of phenolic molecules (Kušić et al. 2006). Moreover, the addition of UV light to the Fe-ZSM-5/H_2_O_2_ process enhances the mineralization of pollutants due to a higher production of radicals and the direct photolysis promoted by the light (Kušić et al. 2006).

On the other hand, caffeine, which is an active compound in some pharmaceuticals for dealing with headaches and a vital constituent of food items, has been treated using zeolite-supported iron nanoparticles. The analysis of the treated caffeine samples revealed neglectable leaching of iron from the catalyst surface, and when the catalyst was reused in the treatment of three successive runs, very similar caffeine removals were observed during the reuse cycles (~ 95%). The heterogeneous catalysis mechanism involved the reaction of iron (II) and iron (III) species on the catalyst surface with hydrogen peroxide to produce the radicals responsible for the caffeine degradation (Anis and Haydar 2019).

On the other hand, iron-supported ZSM-5 was synthesized by the impregnation method, and this material was used to degrade the analgesic ketoprofen (Azusano et al. 2020). The effect of different Fe loading on the zeolite was evaluated (iron loading of 1.015 wt% and 3.569 wt% was considered). The combination of the iron-supported zeolites with hydrogen peroxide led to a complete degradation of the target pharmaceutical at short treatment times (20–35 min), being faster the ketoprofen elimination for the zeolite having the highest iron load. As the zeolites-based Fenton systems may include the homogenous pathway, the leached iron in the solution was measured. The iron etching tests showed that percentages were lower than 15% of the initial loading. Less than 2 mg L^−1^ of iron was detected in the solution (which is the limit concentration allowed by European directives), indicating the participation of the homogeneous mechanism in the system. The supporting of iron on the zeolites to perform a heterogeneous Fenton reaction is an important factor that controls the iron ions amount in solution, which represents an advantage over the homogeneous Fenton systems that may require an additional step to remove formed iron sludges and accomplish the iron limit standards for effluents (Azusano et al. 2020).

**Fig. 5:**
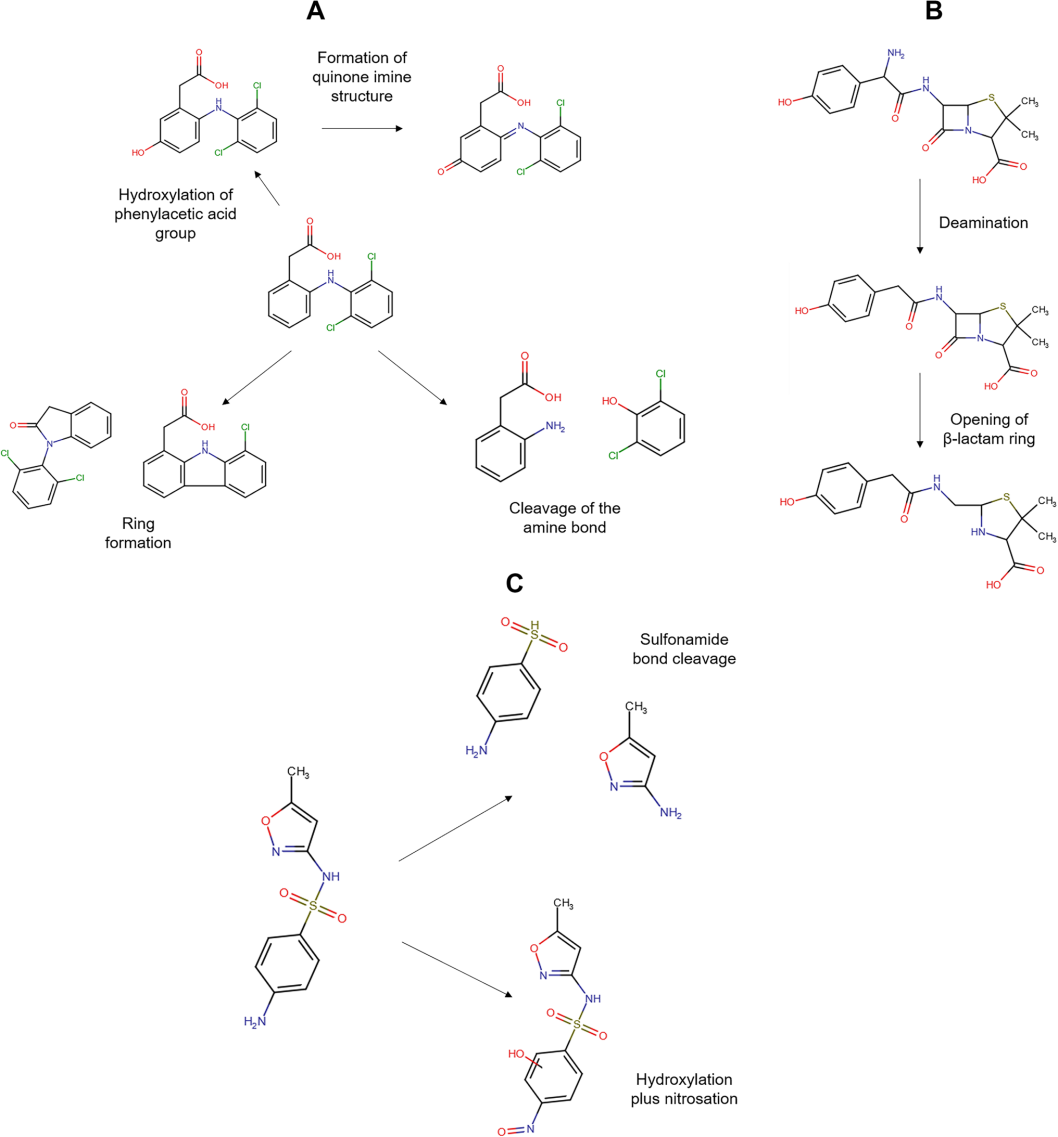
Primary transformations of some representative pharmaceuticals under Fenton-based processes using zeolites. **A** Diclofenac (DFC, figure done by the review authors based on information from Perisic et al. 2016; Salaeh et al. 2016)). **B** Amoxicillin (figure done by the review authors based on information from Jalali et al. (2021)). **C** Sulfamethoxazole (figure done by the review authors based on information from Wang et al. (2020))

In addition to the Fenton system, the photo-Fenton process using zeolites has been applied to deal with pharmaceuticals. Diclofenac has been degraded through the UV-A/Fe-ZSM-5/H_2_O_2_ process. Both the adsorption of the pollutant on the zeolitic material and oxidative degradation contributed to the treatment effectiveness. The oxidative route starts with the HO• action by means of two possible pathways: hydroxylation of the phenylacetic acid group plus the subsequent formation of quinone imine structure and the hydroxylation of bi-phenyl amino moiety, which undergoes C-N bond cleavage in the next step (Fig. 5A). The successive attacks of radicals on the primary intermediates generate small by-products. The by-products having carboxylic acids may bond Fe(III) at the zeolite surface (Eq. 15), and such surface complexes can be degraded upon UV-A illumination (i.e., a photo-reduction reaction, Eq. 16). The treatment of diclofenac by the UV-A/Fe-ZSM-5/H_2_O_2_ system for 120 min led to an improvement in biodegradability and a toxicity decrease of the pharmaceutical solution. Furthermore, the zeolitic catalyst showed good stability (low iron leaching, less than 0.4%) and retained high activity after five consecutive runs. Besides, the washing of the catalyst to remove the organic load before the reuse cycles benefits the performance of the catalyst in the process (Perisic et al. 2016).

15 $$RCOOH+={Fe}^{3+}\to [={Fe}^{3+}-RCOOH{]}_{complex}$$

16 $$[={Fe}^{3+}-RCOOH{]}_{complex}+UVA\to ={Fe}^{2+}+R\cdot +{H}^{+}+{CO}_{2}$$

The ZSM-5 zeolite impregnated with iron species (i.e., iron oxide clusters) has also been employed in a heterogeneous and ultrasound-assisted electro-Fenton (HUEF) for degrading phenazopyridine (an analgesic used to relieve the pain caused by infections or irritations of the urinary tract) (Rostamizadeh et al. 2019). In the electro-Fenton system, the hydrogen peroxide is generated in situ through a cathodic reduction of the dissolved oxygen (Eq. 17). Some ferric species released from the zeolite can be cathodically converted into ferrous ones (Eq. 18). In turn, in the ultrasound system, the waves (represented by “)))”) can facilitate mass transport and promote the generation of extra hydroxyl radicals by the homolytic cleavage of water (Eq. 19) through the acoustic cavitation phenomenon. Then, in the HUEF system, efficient degradation of the target pharmaceutical is obtained by the action of the Fenton process, which is improved by the simultaneous presence of the electrochemical and sonochemical components of the system (Eqs. 17–19). Additionally, the ultrasound waves can also induce a cleaning of the surface and pores of the zeolite and increase the exit of the pollutant and oxidized intermediates from pores. Thus, the HUEF process can degrade ~ 90% of phenazopyridine in only 30 min of treatment, even after a third reuse cycle of the Fe-zeolite (which was regenerated, before the reuse, at 550 °C for 6 h, to remove the retained organic substances from the active sites) (Rostamizadeh et al. 2019).

17 $${O}_{2}+{2H}^{+}+2e-(cathode)\to {H}_{2}{O}_{2}$$

18 $${Fe}^{3+}+1e-(cathode)\to {Fe}^{2+}$$

19 $${H}_{2}O+)))\to H\cdot +HO\cdot$$

Other iron species, such as γ-Fe_2_O_3_, have been composited with NaY zeolite, forming γ-Fe_2_O_3_@NaY, and utilized to eliminate the antibiotic ceftriaxone in water by a photo-catalytic process using UVC light. The system γ-Fe_2_O_3_@NaY/H_2_O_2_/UVC was superior to the individual subsystems (e.g., γ-Fe_2_O_3_@NaY or UVC alone) for degrading the pharmaceutical due to the formation of the hydroxyl radical. From the semiconductor behavior of the iron oxide@zeolite composite in the hydrogen peroxide presence (Eqs. 13–14) and the photolysis of H_2_O_2_ (Eq. 10), the radical species are formed. It is relevant to say that the composite is magnetic, which makes it easily separable/recoverable from aquatic environments by a magnet. Indeed, this strategy is applied to the reuse cycles. Therefore, before reuse, the zeolitic composite is recovered from the treated sample using a hand magnet and washed with distilled water and ethanol, then dried (100 °C for 40 min). After the fifth reuse cycle, the removal of ceftriaxone decreased moderately (first cycle removal, 99%; final cycle elimination, 81%) (Takdastan et al. 2020).

Other semiconductors such as TiO_2_ can also be incorporated into the iron-loaded zeolites. Thus, a composite of TiO_2_-FeZSM-5 in combination with UV light (provided by a Xe lamp) and H_2_O_2_ has been developed for the elimination of diclofenac. The pharmaceutical removal routes involved adsorption on the material (via electrostatic attractions and complexation with = Fe^3+^) with the degradation by generated ROS coming from the interaction of the hydrogen peroxide with iron and light, plus the TiO_2_-photocatalysis. The contribution of homogeneous Fenton was low due to a negligible leaching of iron ions. The action of the formed radical species produced some transformations on diclofenac (Fig. 5A), which subsequently led to short-chain molecules (small dechlorinated structures) that are mainly biodegradable and non-toxic substances (Salaeh et al. 2016).

As an alternative to iron species or TiO_2_, MgO has also been supported on a zeolite. The MgO-zeolite Y material was employed to catalyze the H_2_O_2_ decomposition into radicals for degrading the antibiotic amoxicillin in aqueous matrices. Despite the mechanisms of HO• generation from the interaction of MgO-zeolite Y with H_2_O_2_ remains unclear, the participation of radicals in the amoxicillin degradation was evidenced through the use of scavengers (methanol and isopropyl alcohol) and the elucidation of primary transformations (Fig. 5B). This material has high catalytic activity, achieving up to ~ 80% amoxicillin removal after six reuse cycles. Furthermore, the MgO-zeolite Y/H_2_O_2_ process was able to achieve degradations of amoxicillin higher than 70% after 60 min of treatment, even in complex matrices such as urine or wastewater (Jalali et al. 2021).

Another strategy in the Fenton-based processes to degrade pharmaceuticals using zeolites as catalysts is the replacement of H_2_O_2_ with peracetic acid (CH_3_CO_3_H, PAA). The antibiotic sulfamethoxazole is degraded by an iron-zeolite Y/PAA system. In such a system, the production of hydroxyl radicals involved an initial electron transfer (Eq. 20), followed by the generation of organic radicals (Eqs. 21–23) that could also participate in the elimination of pollutants. In that work, it was evidenced the superior results of Fe^2+^-zeolite Y/PAA compared to Fe^3+^-zeolite Y/PAA, indicating that the ferrous ion on the zeolite is the main activating species for PAA (Wang et al. 2020). The authors reported the presence of sulfate and nitrate anions, or calcium and manganese cations. It was shown a low influence on pharmaceutical degradation, whereas a negative effect was found at low concentrations of chloride and carbonate anions. However, at high concentrations of CO_3_^2−^, positive effects on degradation are observed due to an abundant generation of carbonate radicals (which have longer half-life time than HO•, and they also are degrading species) through the reaction of carbonate with hydroxyl radicals. In turn, natural organic matter strongly affected the degradation of sulfamethoxazole because of the competition by the radicals (Wang et al. 2020).

20 $$={Fe}^{2+}+{CH}_{3}{CO}_{3}H\to ={Fe}^{3+}+{CH}_{3}{COO}^{-}+HO\cdot$$

21 $$={Fe}^{3+}+{CH}_{3}{CO}_{3}H\to ={Fe}^{2+}+{CH}_{3}{CO}_{3}\cdot +{H}^{+}$$

22 $$={Fe}^{2+}+{CH}_{3}{CO}_{3}H\to ={Fe}^{3+}+{CH}_{3}COO\cdot +{HO}^{-}$$

23 $${CH}_{3}COO\cdot \to {CH}_{3}\cdot +{CO}_{2}$$

As mentioned above, radicals are the main ones responsible for the degradation of sulfamethoxazole. The transformations induced by such degrading agents have been also assessed. The attacks of HO• on the pharmaceutical initially lead to hydroxylation of the aromatic ring plus nitrosation, in addition to the cleavage of the sulfonamide bond (S–N) (Wang et al. 2020). Figure 4C shows the primary transformations of sulfamethoxazole under Fenton-based processes using zeolite-derived catalysts.

In addition to the transformations of the pollutants by the radical species, operational parameters such as the catalyst dose, pH, and zeolitic material/peroxide concentration are commonly evaluated in the Fenton-based processes (Table 3). It can be mentioned that the increase of the zeolitic catalyst dose typically enhances the elimination of pharmaceuticals, which is associated with higher availability of the active sites for adsorption and/or decomposition of peroxides into radicals (Perisic et al. 2016; Takdastan et al. 2020). Also, pH is a determinant parameter for the Fenton-based process performance. Acidic pHs (e.g., 2.8–4.0) are preferred due to the formation of ferric hydroxides which is favorable at circumneutral pH values, which limits iron to participating either in the catalytic cycle or to form active complexes that favor the degradation of pollutants (Perisic et al. 2016). However, in some cases, the increase in the pH can promote attractive interactions between the pharmaceutical and the zeolitic material surface (Rostamizadeh et al. 2019). Besides, the increase in the initial H_2_O_2_ concentration, the degradation and mineralization efficiency of the studied system was also improved, but only up to a certain level, where a further increase in the peroxide concentration was inefficient. Moreover, the system efficiency decreases due to a hydrogen peroxide excess because of an auto-scavenger effect (i.e., the reaction of HO• with the H_2_O_2_ to form weaker radicals such as HOO•) (Kušić et al. 2006; Takdastan et al. 2020).

**Table 3:** Degradation of pharmaceuticals by Fenton-based process using zeolites

Pharmaceutical	System	Main result of parameter effects	References
Phenol (moiety very common in the pharmaceuticals)	Fe-ZSM5/H_2_O_2_	The highest pollutant mineralization is achieved at Fe-ZSM5/H_2_O_2_ ratio of 1:40 and Fe-ZSM5 dosage of 1.51 g L^−1^ (pH: 3.0)	Kušić et al. (2006)
Caffeine	Zeolite-supported iron nanoparticles/H_2_O_2_	Pharmaceutical removal > 80% is achieved when the pH is in the range of 5.8–7.0 and Fe/H_2_O_2_ ratio of 2.5–3.0	Anis and Haydar (2019)
Ketoprofen	Iron-supported ZSM5/H_2_O_2_	Catalyst loading is varied between 1 and 4 g L^−1^ (pH: 2.8–3.0), the pharmaceutical degradation is higher as the dose of the zeolitic material increased	Azusano et al. (2020)
Diclofenac	UV-A/Fe-ZSM5/H_2_O_2_	Optimization of parameters using an experimental design and response surface methodology. Favorable conditions are pH: 4, H_2_O_2_: 50 mmol L^−1^, and zeolite dose: 2.0 mmol L^−1^ (as the Fe concentration)	Perisic et al. (2016)
Phenazopyridine	Ultrasound-assisted electro-Fenton using an iron oxides-ZMS5 catalyst	The optimum operating conditions are pH: 7, current of 100 mA, zeolitic catalyst: 0.2 g L^−1^, and ultrasonic power density 600 W L^−1^	Rostamizadeh et al. (2019)
Ceftriaxone	γ-Fe_2_O_3_@NaY/H_2_O_2_/UVC	Efficient degradation at pH: 4.0, catalyst dose: 1.17 g L^−1^, H_2_O_2_: 30 mmol L^−1^, and presence of UVC light	Takdastan et al. (2020)
Amoxicillin	MgO-Zeolite Y/H_2_O_2_	The best removal efficiency in distilled water is achieved at acidic pH (5.0), H_2_O_2_: 0.1 mL/100 mL, and catalyst: 7 g L^−1^. Good process performance in complex matrices (urine, wastewater, and urban water) showing amoxicillin degradations > 70%	Jalali et al. (2021)
Sulfamethoxazole	Fe^3+^-zeolite Y/peracetic acid (PAA)	Proper degradation is obtained at pH 7.0, catalyst: 0.8 g L^−1^, and PAA: 0.4 mmol L^−1^. The presence of Cl^−^ or SO_4_^2−^ (even at high concentrations, e.g., 1.0 mmol L^−1^) did not affect the pharmaceutical degradation	Wang et al. (2020)

### Activation of persulfate using zeolites

Persulfates (i.e., peroxydisulfate and peroxymonosulfate) can be activated by zeolitic structures containing cobalt ions, cobalt oxides, iron ions, zero-valent iron (ZVI), or iron oxides (Ushani et al. 2020; Kohantorabi et al. 2021; Wang et al. 2022). The interaction of peroxydisulfate (PDS, S_2_O_8_^2−^) or peroxymonosulfate (PMS, HSO_5_^−^) with these kinds of materials involves electron transfer mechanisms to produce radical species, as illustrated in Eqs. 24–29 (Cong et al. 2017; Wang et al. 2022), where the presence of Co^2+^/Co^3+^ and Fe^2+^/Fe^3+^ cycles favors the catalytic performances (Kohantorabi et al. 2021).

24 $${Co}^{2+}@zeolite+{{HSO}_{5}}^{-}\to {Co}^{3+}@zeolite+{SO}_{4}\cdot^{-}+{HO}^{-}$$

25 $${Co}^{3+}@zeolite+{{HSO}_{5}}^{-}\to {Co}^{2+}@zeolite+{SO}_{5}\cdot^{-} +{H}^{+}$$

26 $${Fe}^{0}@zeolite+{2S}_{2}{{O}_{8}}^{2-}\to {Fe}^{2+}@zeolite+{2SO}_{4}\cdot^{-}+{{2SO}_{4}}^{2-}$$

27 $${Fe}^{2+}@zeolite+{S}_{2}{{O}_{8}}^{2-}\to {Fe}^{3+}@zeolite+{SO}_{4}\cdot^{-}+{{SO}_{4}}^{2-}$$

28 $${Fe}^{3+}@zeolite+{S}_{2}{{O}_{8}}^{2-}\to {Fe}^{2+}@zeolite+{S}_{2}{O}_{8}\cdot^{-}$$

29 $${Fe}^{0}@zeolite+{Fe}^{3+}@zeolite\to {2Fe}^{2+}@zeolite$$

The action of SO_4_• − coming from the interaction of zeolitic materials with PDS has been evidenced in the treatment of the pharmaceutical tetracycline. An ash-derived zeolite coated with Fe_3_O_4_ (MIZ, which is magnetic) was shown to be a good heterogeneous catalyst for the PDS activation toward the degradation of the target antibiotic. The MIZ/PDS process achieved an efficient and fast tetracycline elimination. Moreover, the catalyst had high stability according to the good results in the recycling experiments and the leaching test for iron (Zhao et al. 2023).

**Fig. 6:**
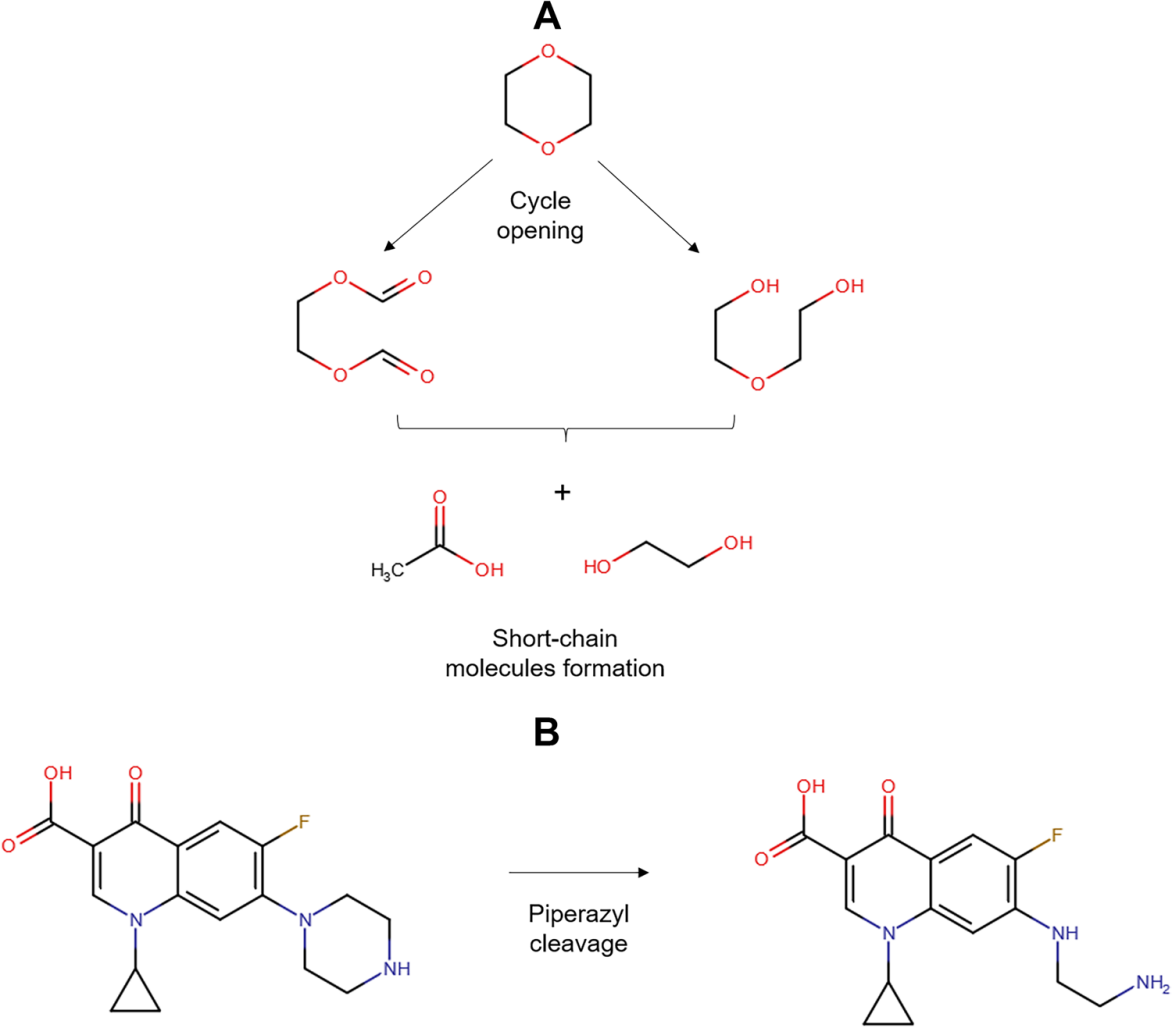
Initial transformations of dioxane via attacks of radicals (**A**) and ciprofloxacin through reaction with singlet oxygen (**B**) during the activation of persulfates by zeolitic catalysts. This figure was made by the review authors using information from Serna-Galvis et al. (2023) and Malkapuram et al. (2023)

PDS has also been activated by an iron nanoparticle decorated zeolite (FeNP-Z) with the assistance of ultrasound (at 24 kHz and 105 W cm^−2^) to degrade 1,4-Dioxane, which is a ubiquitous water pollutant. This is found as a trace element in personal care products and it is also used as a purifying agent in pharmaceutical manufacturing. The degradation of 1,4-Dioxane by the FeNP-Z/PDS/ultrasound system was superior to the action of PDS/ultrasound or ultrasound alone. In the FeNP-Z/PDS/ultrasound system, the elimination of 1,4-Dioxane was associated with attacks of radicals, coming from the combined effects of the persulfate activation by the zeolitic catalyst, and the PDS peroxide bond cleavage by ultrasound (Eq. 30). Also, it can be considered that the heterogeneous catalyst acts as cavitation nuclei. Furthermore, the disaggregation of the catalyst particles by ultrasound waves leads to a greater available surface area of the catalyst, favoring the reactants to interact with each other more easily (Malkapuram et al. 2023). It is important to note that the produced radicals induced oxidations via cyclic structure openings, with the subsequent formation of small molecules, such as ethylene glycol and acetic acid (Fig. 6A).

30 $${S}_{2}{{O}_{8}}^{2-}+)))\to {2SO}_{4}\cdot^{-}$$

PDS has been also activated by a zeolite-TiO_2_ composite under UVA irradiation for degrading the mixture of the pharmaceuticals cefixime and phenazopyridine in the presence of a dye. In this kind of system, transition metals as impurities present in zeolite and TiO_2_ (e.g., the zeolite used therein contains Fe_2_O_3_ at 1.3%) activate persulfate, forming sulfate radicals (Eqs. 27 and 28). The introduction of UVA light increases the elimination of pollutants because of the action of TiO_2_ as a semiconductor (Eskandarian et al. 2016). The irradiation of zeolite-TiO_2_ composites with UVA light promotes an electron from the filled valence band to the vacant conduction band and leaves a hole in the valence band, thus forming the h + /e − pairs (Eq. 31). The photogenerated hole is strongly oxidizing, and it leads to the generation of HO• from water (Eq. 32). In turn, the electron in the conduction band is a highly reducing agent that induces the reduction of dissolved oxygen, producing superoxide radical anion and hydroperoxyl radical (Eqs. 33 and 34). Also, the electron in the conduction can activate PDS (which acts as an electron acceptor), leading to the sulfate radical formation (Eq. 35). Moreover, the interaction with PDS (Eq. 35) decreases the electron–hole recombination, thus favoring the formation of the radicals and, consequently, the degradation processes (Pan et al. 2014).

31 $${TiO}_{2}@zeolite+{UV}_{light}\to {TiO}_{2}@zeolite(h+/e-)$$

32 $${h}^{+}+{H}_{2}O\to HO\cdot +{H}^{+}$$

33 $${e}^{-}+{O}_{2}\to {O}_{2}\cdot^{-}$$

34 $${O}_{2}\cdot^{-} +{H}^{+}\to HOO\cdot$$

35 $${e}^{-}+{S}_{2}{{O}_{8}}^{2-}\to {SO}_{4}\cdot^{-}+{{SO}_{4}}^{2-}$$

Another option to activate persulfates and eliminate organic compounds in water matrices involves zeolites and heating (Huang et al. 2020; Qian et al. 2022). Guaiacol (2-methoxyphenol), which is an important secondary standard and chemical precursor widely used in pharmaceutical industries, has been degraded by activated PDS with an iron-modified clinoptilolite plus heat. Ferrous ions adsorbed on the zeolite promoted better generation of radicals and guaiacol degradation by the effect of a slow and continuous iron release from the zeolite compared to the directly activated persulfate process by ferrous sulfate (fast and short-life activation). Furthermore, the heating of the Fe^2+^-zeolite/PDS system led to a synergistic degradation of the pollutant, due to the thermal energy (represented by Δ) also cleavages the persulfate to generate SO_4_• − (Eq. 36). Hence, a fast elimination and mineralization of guaiacol was achieved, showing high degradation efficiency even after the third reuse cycle of the zeolitic catalyst (Huang et al. 2020).

36 $${S}_{2}{{O}_{8}}^{2-}+\Delta \to {2SO}_{4}\cdot^{-}$$

In addition to PDS, the peroxymonosulfate anion can also be activated using zeolitic materials. A zeolite 4A-type activated PMS toward non-radical species (i.e., singlet oxygen) for dealing with ciprofloxacin. This zeolite was able to activate PMS but no PDS or H_2_O_2_. It is proposed that the activation is triggered by an acid–base interaction between deprotonated terminal oxygens on the zeolite surface and PMS, as represented by Eqs. 37–38. The singlet oxygen generated in the zeolite 4A/PMS process attacks the antibiotic modifying its piperazyl ring (Fig. 6B). The zeolite 4A/PMS system induced a fast degradation of the pharmaceutical in a simulated urine matrix. Moreover, the catalyst activated PMS even after three reuse cycles without the involvement of the common recovery steps (filtration, washing, and drying) for a solid material (Serna-Galvis et al. 2023).

37 $$\left(Zeolite\right)-{O}^{-}+H-{{SO}_{5}}^{-}\to \left(Zeolite\right)-OH+{{SO}_{5}}^{-}$$

38 $${{SO}_{5}}^{2-}+{{HSO}_{5}}^{-}\to {{HSO}_{4}}^{-}+{{SO}_{4}}^{2-}+{ }^{1}{O}_{2}$$

Cobalt-containing zeolite imidazolium frameworks (ZIFs), such as ZIF-67, ZIF-12, and ZIF-9, also have great potential for the activation of PMS (Kohantorabi et al. 2021). ZIFs consist of metal–organic frameworks, i.e., hybrid materials built from organic ligands and metal ions, having high surface area, controllable pore structures, multiple functionalities, and high thermal and chemical resistance. ZIFs are ordered metal ions/cluster arrays linked to organic ligands that form hierarchical porous presenting zeolitic structures (e.g., SOD and RHO topologies) (Cong et al. 2017). Moreover, the use of ZIF heterogeneous catalysts is beneficial in preventing secondary contamination by limiting the uncontrolled leach of homogeneous catalysts to the aqueous solution (Zhang et al. 2022).

ZIF-based materials are very promising heterogeneous catalysts to activate persulfates to degrade organic contaminants (Cong et al. 2017; Zhang et al. 2022). For instance, CuFe_2_O_4_/ZIF-67, which is a magnetic material, was applied to activate PDS for the degradation of the antibiotic tetracycline. It is important to remember that this material has cobalt in its metal-imidazolium framework (metal with highly proven activating capability toward persulfates). The pharmaceutical degradation was only 5% by PS without the catalyst presence, and the removal by the CuFe_2_O_4_/ZIF-67 alone was ~ 22%. The tetracycline degradation by the combination of PDS and CuFe_2_O_4_/ZIF-67 was 92% within 30 min by the main action of the sulfate radical (demonstrated by degradation inhibition with scavengers), having a synergistic effect among Co^3+^/Co^2+^, Cu^2+^/Cu^+^, and Fe^3+^/Fe^2+^ for the PDS activation. Additionally, from a practical point of view, the use of CuFe_2_O_4_/ZIF-67 as a catalyst is successful because this material shows low leaching (< 5%) of the active metals (i.e., Co, Fe, or Cu); it can be reused during six cycles, showing tetracycline removals ≥ 78%, and this catalyst is easily recovered from the solution thanks its magnetic properties (Nagshbandi et al. 2023).

In an analogous way to tetracycline, ciprofloxacin (a fluoroquinolone-type antibiotic) has been degraded by a system involving ZIFs and persulfates. In such a case, a nitrogen-doped ZIF-67 (i.e., N@ZIF-67) combined with PMS was utilized. The incorporation of N into the ZIF-67 structure boosted both the efficiency of degradation and stability of the catalyst for recycling purposes. The N@ZIF-67/PMS system led to 100% of the pharmaceutical degradation by the attacks of superoxide anion radical and singlet oxygen mainly, with minor participation of sulfate radical despite the presence of cobalt in the ZIF structure (Nguyen et al. 2022). The generation of these degrading species could be rationalized as a sequence of reactions. The catalyst adsorbs PMS via hydrogen bonding and releases a sulfur pentoxide anion (Eq. 39), which reacts with water producing H_2_O_2_ (Eq. 40). The hydrogen peroxide is subsequently decomposed by several steps into several ROS (O_2_•^−^, ^1^O_2_, SO_4_•^− ^, and HO•, Eqs. 41–46).

39 $$N@ZIF-67\dots {{HSO}_{5}}^{-}\to N@ZIF-67{H}^{+}+{{SO}_{5}}^{2-}$$

40 $${{SO}_{5}}^{2-}+{H}_{2}O\to {{SO}_{4}}^{2-}+{H}_{2}{O}_{2}$$

41 $$={Co}^{2+}+{{HSO}_{5}}^{-}\to ={Co}^{3+}+{SO}_{4}\cdot^{-}+{HO}^{-}$$

42 $${SO}_{4}\cdot^{-} +{HO}^{-}\to {{SO}_{4}}^{2-}+HO\cdot$$

43 $$HO\cdot +{H}_{2}{O}_{2}\to HOO\cdot +{H}_{2}O$$

44 $$HOO\cdot \to {H}^{+}+{O}_{2}\cdot^{-}$$

45 $${O}_{2}\cdot +HO\cdot \to {}^{1}{O}_{2}+{HO}^{-}$$

46 $$2 {O}_{2}\cdot^{-} +{H}^{+}\to {}^{1}{O}_{2}+{H}_{2}{O}_{2}$$

After seven reuse runs, ciprofloxacin degradation > 90% by the N@ZIF-67/PMS process is achieved, and the amount of cobalt solubilized from the catalyst is very low (< 30 µg L^−1^) in each run, evidencing the good stability of the N@ZIF-67. From the X-ray photoelectron spectroscopy (XPS) analyses, the deconvolution of the Co 2p peak showed a small decrease of Co^2+^/Co^3+^ ratio from 1.55 to 1.38, indicating that, on the surface of N@ZIF-67, a portion of Co^2+^ is converted into Co^3+^ during the activation of PMS (Eq. 41). Furthermore, the effects of different water matrices on degradation were also studied, following the antibiotic removal order: deionized water > tap water > river water. The pharmaceutical degradation was partially inhibited in tap and river water, because of the presence of dissolved organic matter, bicarbonate, sulfate, and chloride anions that scavenge the degrading species. However, the N@ZIF-67/PMS system achieves degradations of ciprofloxacin higher than 80% in tap and river water (Nguyen et al. 2022).

Levofloxacin is another fluoroquinolone-class antibiotic that has been treated using the activation of persulfates by a ZIF. A composite of ZIF-67 with vanadium-titanium magnetite (ZIF-67/VTM) was considered. This catalyst activated PMS toward the production of sulfate radicals by the breakage of the O–O bond of PMS. In PMS activation, the redox cycle of Co in the ZIF structure played a relevant role, and the ZIF-67 shell/VTM core structure is synergistic due to the presence of ferrous/ferric iron species. Those species improve the redox cycle of Co in the catalyst (Eq. 47) plus the PMS activation by = Fe^2+^. The produced radicals efficiently degrade levofloxacin, showing degradations > 99% even after the third reuse cycle, having low Co and iron leaching (< 0.09 mg L^−1^), and the catalyst has an excellent magnetic separation from the solution. Moreover, during the treatment, the presence of nitrate, sulfate, and phosphate acid anions slightly inhibited the levofloxacin degradation, whereas chloride and bicarbonate anions strongly affected the antibiotic elimination (Wan et al. 2022).

47 $$={Co}^{3+}+={Fe}^{2+}\to {Co}^{2+}+{Fe}^{3+}$$

### Environmental impact of the transformation products coming from aots involving zeolites

At this point, it is important to remember that the innocuity of the products generated from the degradation of pharmaceuticals should be assessed to guarantee the positive environmental effects of the applied processes (Salaeh et al. 2016). However, most of the works revised above had little information about the environmental impact of the transformation products. Therefore, considering the limited information about the biodegradability and toxicity of the primary transformation products coming from AOTs that utilize zeolites for degrading pharmaceuticals, an initial contribution to overcoming these lacking topics, using a free predictive tool (i.e., BiodegPred (Garcia-Martin et al. 2020)), is made in this review.

**Fig. 7:**
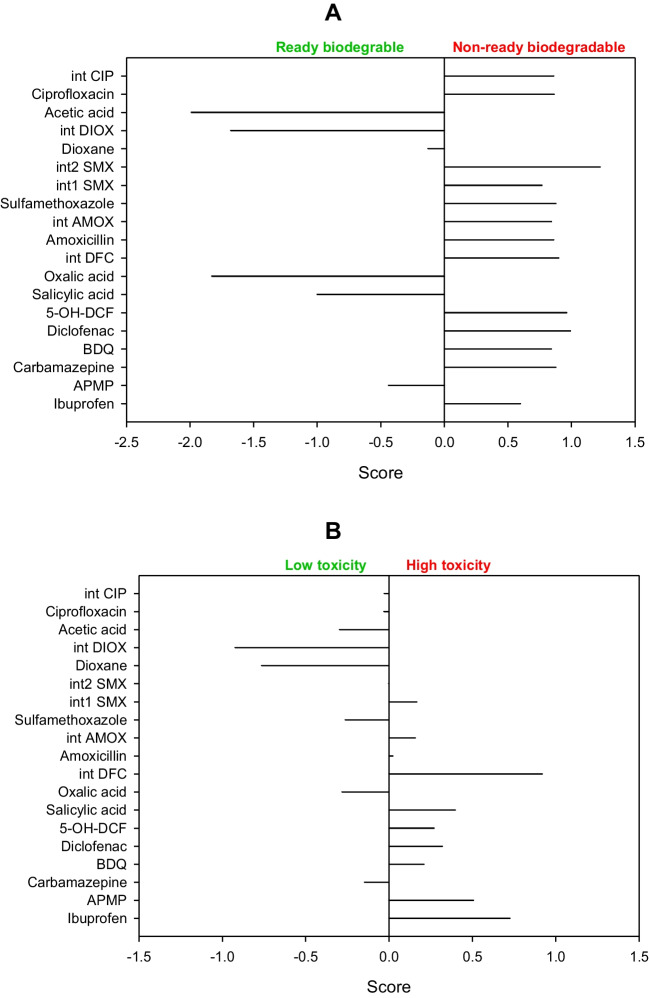
Predicted biodegradability and toxicity of pharmaceuticals and some of their primary transformation products. The predictions were obtained by the review authors, using the free online tool BiodegPred (Garcia-Martin et al. 2020). The *x*-axis refers to the support vector machine (SVM) predictor score for the tested parameter, discriminating between two categories (i.e., “ready biodegradable” and “non-ready biodegradable” for the biodegradability parameter; or “low toxicity” and “high toxicity”; for the toxicity parameter) and using the score = 0 as a reference value for the categories differentiation

Illustrative cases of each revised AOT were considered. Table 4 and Fig. 7 compare the predicted results of biodegradability and toxicity, for the parent pharmaceutical and some of its transformation products, obtained via the BiodegPred tool. It must be mentioned that the biodegradability criterion in BiodegPred is based on a test database (NITE) from the Ministry of International Trade and Industry, Japan, in which the target substance is inoculated and incubated with 30 mg L^−1^ of activated sludge. The biological oxygen demand (BOD) is continuously monitored for 28 days. If the BOD is higher than 60% of the theoretical oxygen demand, the target substance is considered “ready biodegradable”; otherwise, the compound is recognized as “non-ready biodegradable.” In turn, the toxicity criterion in BiodegPred is based on mammalian oral toxicity (usually taken as a proxy for human toxicity), which uses the Pesticide Properties DataBase (PPDB) from the University of Hertfordshire. The toxicity classification of PPDB considers the acute oral LD_50_ in mammals; that is, those substances presenting LD_50_ > 2000 mg/kg have “low toxicity,” and compounds with LD_50_ ≤ 2000 mg/kg exhibit “high toxicity” (Garcia-Martin et al. 2020).

**Table 4:**
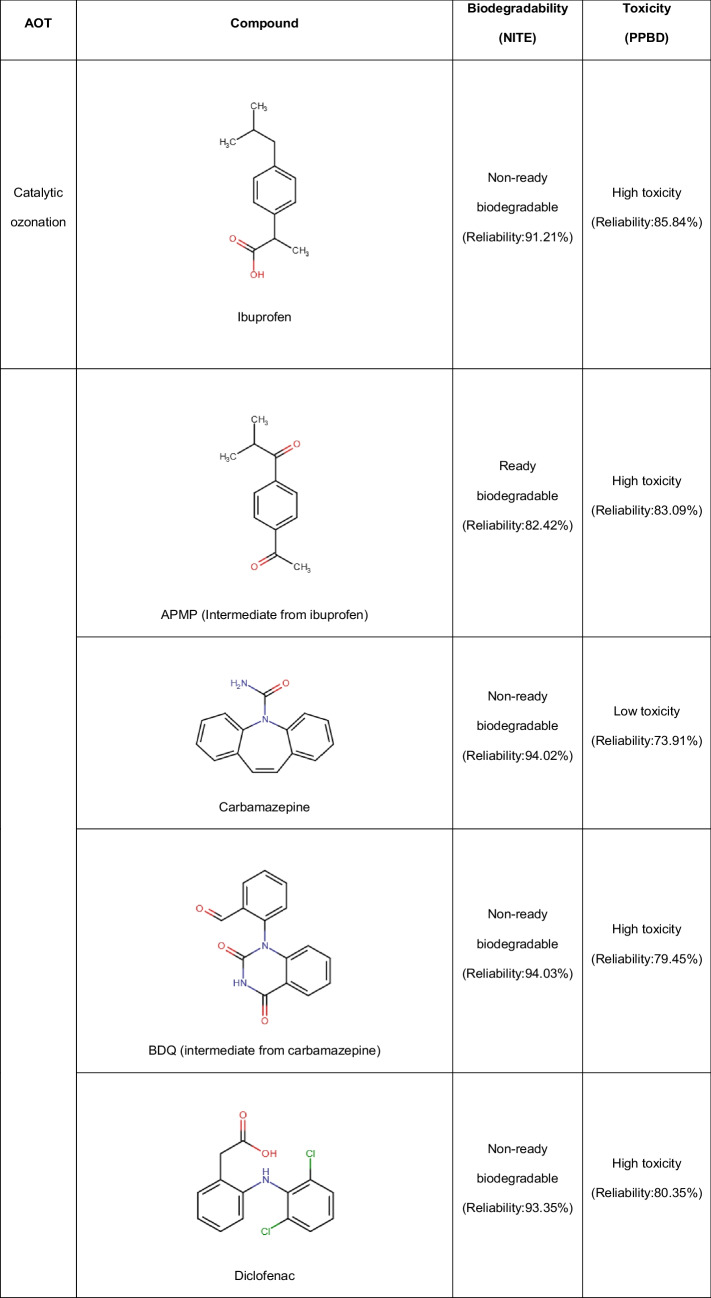

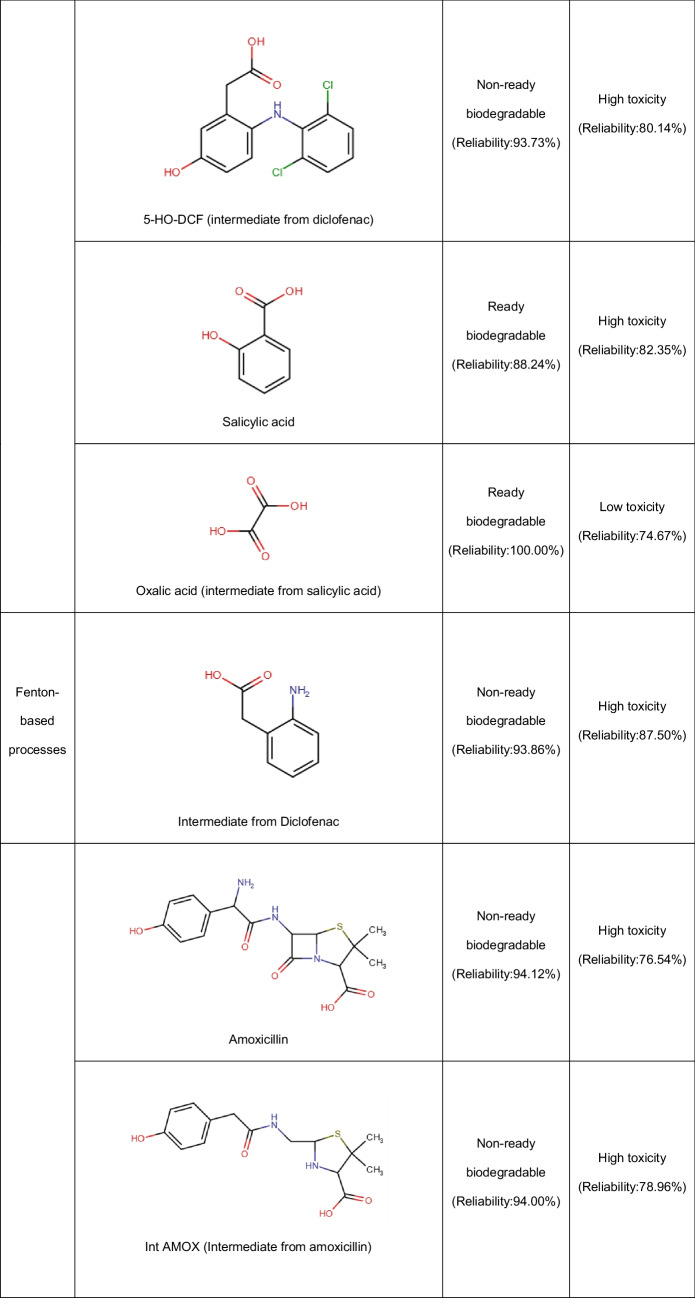

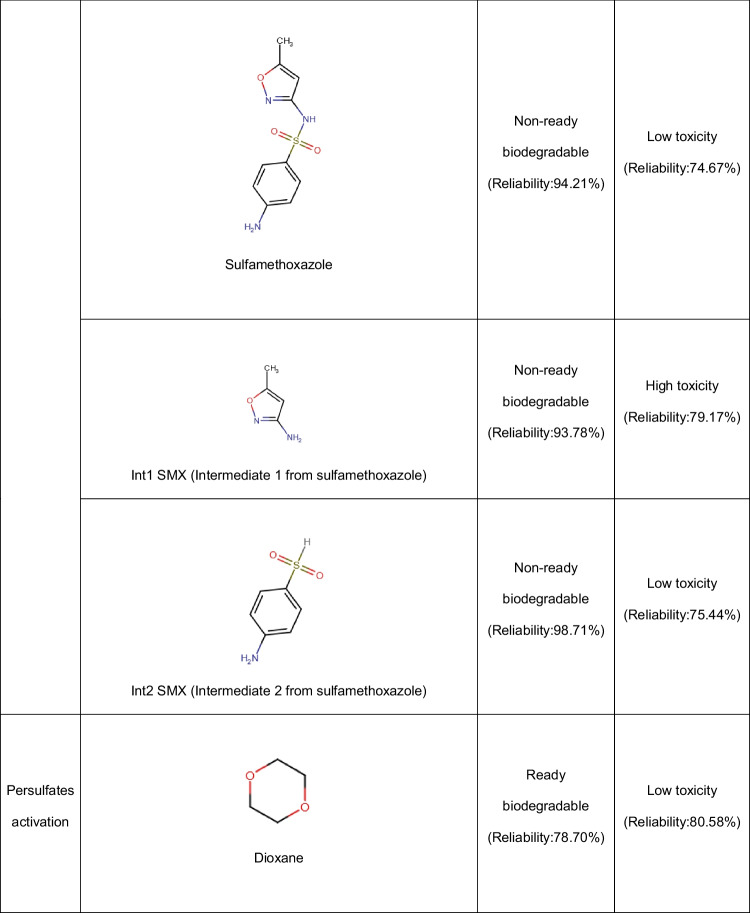

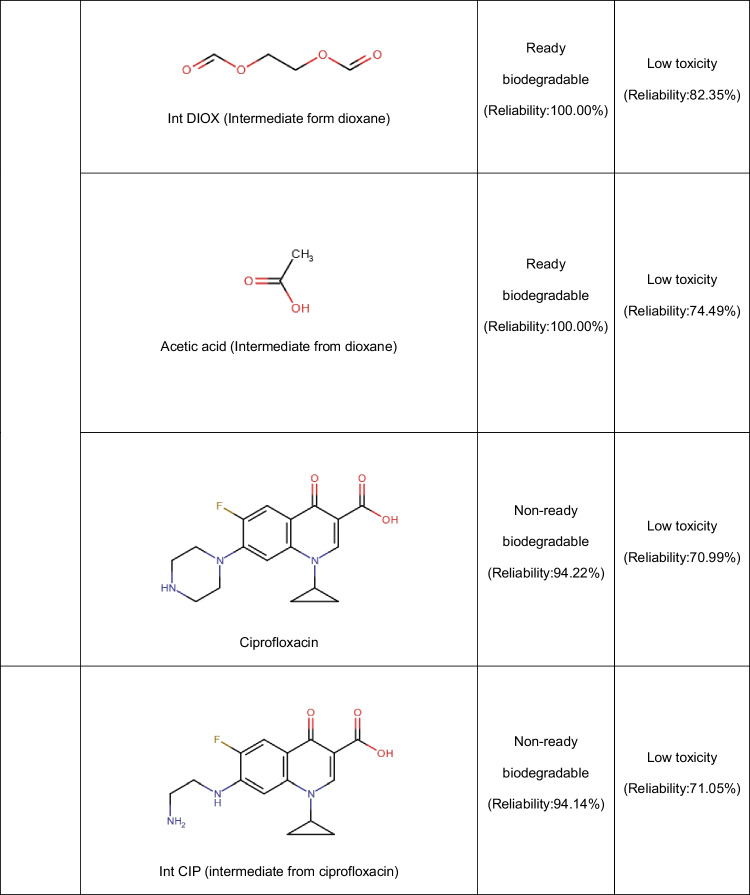
Predicted biodegradability and toxicity for some pharmaceuticals and their primary transformation products*

^*^Biodegradability and biological toxicity can be outlined/predicted based on the chemical structure of the pollutant. As the experimental determinations of biodegradability and toxicity parameters are very expensive and slow, several efforts have been made to predict them using computational/theoretical tools such as BiodegPred, which is a combined platform that provides an informed prognosis of the chance a target compound can be catabolized in the biosphere, and of its toxicity (Garcia-Martin et al. 2020)

We should indicate that the BiodegPred tool uses support vector machine (SVM) predictors, which have been trained to generate scores for the biodegradability or toxicity parameters, allowing us to discriminate between two categories (i.e., “ready biodegradable” and “non-ready biodegradable” for the biodegradability; or “low toxicity” and “high toxicity”; for the toxicity parameter). There is a score of reference (score = 0, named hyperplane) that separates the two categories. Then, scores higher than zero indicate the evaluated compound lies in the “non-ready biodegradable” category for the biodegradability parameter and “high toxicity” for the toxicity parameter. Meanwhile, scores lower than zero mean that the target substance belongs to the “ready biodegradable” category for the biodegradability parameter and “low toxicity” for the toxicity parameter (Garcia-Martin et al. 2020).

From the predicted results for biodegradability and toxicity (Table 4 and Fig. 7), it can be highlighted that those primary degradation products, which have small structural modifications regarding the initial pharmaceutical, have a low change in the biodegradability and toxicity parameters (see the case of ciprofloxacin, Fig. 7), and even in some cases, the primary by-products are less biodegradable or have higher toxicity than the parent compounds (see, for instance, the cases of carbamazepine or sulfamethoxazole in Fig. 7). However, very strong transformations of the pharmaceuticals lead to low-toxic and biodegradable products, such as short-chain aliphatic acids (e.g., oxalic acid or acetic acid, Fig. 7). Indeed, these short-chain degradation products are widely recognized in the literature as biodegradable and low-toxic compounds (Salaeh et al. 2016).

The theoretical analyses presented herein (Table 4 and Fig. 7) plus some experimental works (Salaeh et al. 2016) denote the relevance of extending the treatment time of AOT application up to the achievement of biodegradable and non-toxic products or even the mineralization. This is a critical point because some AOTs using zeolites do not always lead to the complete mineralization of pharmaceuticals, or in other cases, the processes are stopped when 100% of the pharmaceutical concentration decay and environmental concern substances may be produced. On the other hand, it must be highlighted that tools such as BiodegPred are a good approach to obtaining initial information about the environmental impact of the transformation products. Nevertheless, all the predicted results must be verified and validated through experimental work.

## Conclusions

The adsorption of pharmaceuticals has been widely performed using natural and synthetic zeolites. This kind of process strongly depends on the framework of the zeolites. For instance, zeolites having large micropore volumes or surface areas show high capacities for the adsorption of organic pollutants. It is important to mention that the adsorption of organic pollutants as many pharmaceuticals is also influenced by the hydrophobic interactions with the zeolites (such interactions are more common for zeolites with high Si/Al ratios). The presence of metals, ionic liquids, or surfactants in zeolites can drive the adsorption by a combination of hydrophobic and electrostatic interactions. Moreover, the prevailing interaction mechanism depends on pH and the pKa of the target pharmaceutical too.

Zeolitic materials can also effectively activate ozone (O_3_) and peroxide-type compounds (e.g., H_2_O_2_, HSO_5_^−^, S_2_O_8_^2−^, or CH_3_CO_3_H) for the generation of reactive degrading species (forming AOTs), where the presence in these materials of transition metals such as Fe, Co, Cu, Pt, Pd, or even semiconductor such iron oxides and TiO_2_ plays a determinant role during the activation process. Therefore, the leaching of the transition metals from the zeolitic catalysts to the water sample must be assessed (we should mention that this evaluation is missed in some studies), because such a test gives useful information about the collateral undesired pollution by metals, stability, and reusability of the material in the applications of AOTs.

On the other hand, the zeolite-based advanced oxidation processes promote the degradation of pharmaceuticals and can improve the regeneration/reuse of zeolites regarding the adsorption process. Besides, the generated strong reactive species (e.g., HO•) by AOTs involving zeolites transform the pharmaceuticals. However, in many cases, there is no mineralization (i.e., a complete conversion of the initial pollutant into carbon dioxide, water, and inorganic ions), forming stable by-products, whose environmental impact must be determined, and from the revised literature, it was evidenced that few studies show the toxic potential and/or the biodegradability of the resultant transformation products and solutions after the treatment of pharmaceuticals by zeolite-based AOTs.

Although theoretical tools can provide a useful initial approach to understanding the biodegradability and toxicity of the primary transformation products coming from the degradation of pharmaceuticals, these parameters also require experimental evaluation and confirmation. Additionally, in the particular case of antibiotics, the testing of antimicrobial activity removal is required to limit the proliferation of antibiotic-resistant bacteria. Finally, we can remark that the evaluation of zeolite-based AOTs in complex matrices is scarce, and future research works should include results for the treatment of pharmaceuticals in diverse actual water matrices to better evidence the applicability and feasibility of these AOTs.

## Data Availability

Data and materials will be available upon request to the authors.
